# Current Status, Challenges, and Possible Solutions of EEG-Based Brain-Computer Interface: A Comprehensive Review

**DOI:** 10.3389/fnbot.2020.00025

**Published:** 2020-06-03

**Authors:** Mamunur Rashid, Norizam Sulaiman, Anwar P. P. Abdul Majeed, Rabiu Muazu Musa, Ahmad Fakhri Ab. Nasir, Bifta Sama Bari, Sabira Khatun

**Affiliations:** ^1^Faculty of Electrical & Electronics Engineering Technology, Universiti Malaysia Pahang, Pekan, Malaysia; ^2^Innovative Manufacturing, Mechatronics and Sports Laboratory, Faculty of Manufacturing and Mechatronic Engineering Technology, Universiti Malaysia Pahang, Pekan, Malaysia; ^3^Centre for Fundamental and Continuing Education, Universiti Malaysia Terengganu, Kuala Nerus, Malaysia

**Keywords:** brain-computer interface (BCI), electroencephalogram (EEG), machine learning, classification, feature extraction

## Abstract

Brain-Computer Interface (BCI), in essence, aims at controlling different assistive devices through the utilization of brain waves. It is worth noting that the application of BCI is not limited to medical applications, and hence, the research in this field has gained due attention. Moreover, the significant number of related publications over the past two decades further indicates the consistent improvements and breakthroughs that have been made in this particular field. Nonetheless, it is also worth mentioning that with these improvements, new challenges are constantly discovered. This article provides a comprehensive review of the state-of-the-art of a complete BCI system. First, a brief overview of electroencephalogram (EEG)-based BCI systems is given. Secondly, a considerable number of popular BCI applications are reviewed in terms of electrophysiological control signals, feature extraction, classification algorithms, and performance evaluation metrics. Finally, the challenges to the recent BCI systems are discussed, and possible solutions to mitigate the issues are recommended.

## Introduction

Communication, or social interaction, is one of the key principles of human civilization. This quality enables one to share emotions, expectations, and creative thoughts amongst human beings. In the event that this communication is established through speech, gesture, or writing, human communication becomes easier and devoid of constraints. Nonetheless, people who are suffering from locked-in syndrome do not have the aforementioned options for interaction. Patients with locked-in syndrome could not interact or express themselves, although they are well-cognizant of things around them (Ashok, 2017). Amyotrophic lateral sclerosis (ALS), cerebral palsy, brain stem stroke, multiple sclerosis, cerebral palsy, and spinal cord injury are the main causes of locked-in syndrome (Holz et al., 2013). It is almost impossible for a person who is affected by the locked-in syndrome to communicate with other persons, and hence, Brain–Computer Interface (BCI) is a promising means to furnish them with basic communication abilities. Fundamentally, the human brain and devices are interfaced through the concept of BCI in which the users will have to generate a variety of brain waves that will be recognized and converted into commands to the devices (Volosyak et al., 2017). In its earlier days, researchers intended to use this technology to develop assistive devices for medical purposes only. Nonetheless, the employment of this technology has expanded, and it has found its way into non-medical applications. It is discernible that over the last 15 years, a considerable number of original articles as well as reviews have been published on BCI. An excellent review article on BCI spelling systems was published in Rezeika et al. (2018), giving a concise description of some successful recent BCI spelling models, including their categories, methodologies, and results. The authors also listed some limitations of the current systems as well as making recommendations for directions that could be pursued to overcome the issues. However, it is worth mentioning that the content of the review emphasized and was restricted to only BCI spellers. There are other notable BCI reviews that cater to specific applications, for instance, wheelchair control (Fernández-Rodríguez et al., 2016; Al-qaysi et al., 2018), BCI mobile robot (Bi et al., 2013), emotion recognition using EEG (Al-Nafjan et al., 2017), biometrics (Del Pozo-Banos et al., 2014; Alariki et al., 2018), and virtual reality and gaming (Kaplan et al., 2013; Ahn et al., 2014; Cattan et al., 2018). Nevertheless, some pertinent information was missing or not duly reported, for instance, descriptions of methodology and evaluation metrics employed, and/or future directions of the research.

Electroencephalogram (EEG) control signals and their classifications have been briefly discussed in an excellent review (Ramadan and Vasilakos, 2017). The authors reviewed state-of-the-art BCI solutions with regards to both hardware and software; however, it was noticeable that the applications, as well as the signal processing methods, were not taken into consideration. Likewise, Hwang et al. (2013) have summarized articles related to EEG-based BCI systems published from 2007 to 2011. Notwithstanding, the review did not entirely reflect the current state-of-the-art and did not provide any future directions for the research. Conversely, in Abdulkader et al. (2015), the fundamental aspects that cover the wide spectrum of EEG-based BCI systems were reviewed; however, the number of articles reviewed was rather limited. Lotte et al. (2007) provided a review of the classification algorithms used in EEG-based BCI systems up to 2006. In their second review (Lotte et al., 2018), the application of machine learning algorithms used on BCI systems from 2007 to 2017 was reviewed. In both articles, the authors surveyed EEG control signals, features, classification methods, and classification accuracy. Moreover, the authors provided some guidelines on selecting the appropriate classification algorithms; nonetheless, the articles lacked evaluation of the performance metrics. A review on portable and non-invasive modalities such as EEG-, functional transcranial Doppler (fTCD)-, and near-infrared spectroscopy (NIRS)-based hybrid BCI was reported in Banville and Falk (2016). Twenty-two items were investigated from 55 journal articles published between 2008 and 2014. The authors reviewed non-invasive modalities, EEG control signal, experiment protocol, signal processing methods, and system evaluation, as well as shedding some light on future directions for EEG-based BCI research. However, a comparison of the experimental results between the BCI applications or EEG modalities were not made available, and a similar observation was also noticed in Abiri et al. (2019).

Therefore, the objectives of this article are to review EEG-based BCI systems with regards to the different brain control signals, feature extraction methods, classification algorithms, and evaluation metrics utilized. Moreover, a concise overview of EEG-based BCI systems is presented here so that the reader(s) may select the most appropriate method for a specific BCI system. In addition, related research gaps that warrant further exploration are also presented in this paper. Of note, salient problems associated with EEG-based BCI systems are listed in terms of its applications, and possible solutions to mitigate the issues are also recommended. Moreover, this review, unlike other published review articles with regards to EEG-based BCI systems that were specific in nature, particularly with respect to either its specific applications or part of the methodology employed (e.g., feature extraction, signal processing, and classification, amongst others), provides a more comprehensive overview that can easily be comprehended by the readers to identify the gaps in the body of knowledge. This article is structured in the following manner: section Essential Components of BCI Technology presents a brief discussion on BCI overview, section Popular EEG Based BCI Applications Aspect illustrates the review of popular EEG-based BCI applications, section Current Challenges and Directions discusses the challenges, giving recommendations, section Conclusion draws the conclusion of the present review paper.

## Essential Components of BCI Technology

**Figure 1:**
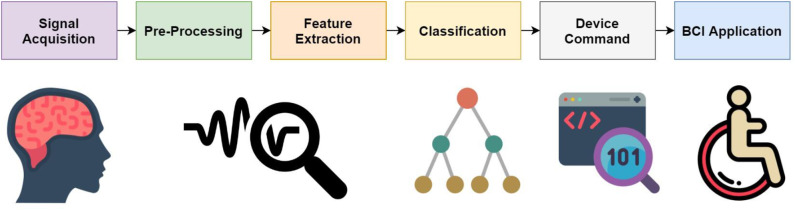
General architecture of a brain-computer interface.

Brain–Computer Interface (BCI) is an effective as well as a powerful tool for user-system communication. Through this system, from the issuance of the commands to the completion of the interaction, no external devices or muscle intervention is required (Van Erp et al., 2012). Nicolas-Alonso and Gomez-Gil (2012) defined brain–computer interface (BCI) or brain–machine interface (BMI) as a hardware and software communications strategy that empowers humans to interact with their surroundings with no inclusion of peripheral nerves or muscles by utilizing control signals produced from electroencephalographic activity. Every BCI system essentially consists of five components: brain activity measurement, preprocessing, feature extraction, classification, and translation into a command (Mason and Birch, 2003). Figure 1 depicts a typical block diagram that illustrates the different stages of EEG signal processing for BCI. In the brain activity acquisition phase, the brain activity from the targeted user is captured through the various types of EEG sensors (Wolpaw et al., 2006). The raw EEG data includes a variety of artifacts, and these artifacts are eliminated in the pre-processing phase (Bashashati et al., 2007a). Feature extraction aims at describing the signals by a few relevant values called “features;” often, at this stage, the selection of significant features is also investigated (Bashashati et al., 2007a). The extracted features are then classified through different machine learning and deep learning algorithms in the classification phase (Lotte et al., 2007). Finally, the classified outcomes are translated into device commands to develop real-life BCI application (Kubler et al., 2006).

### Branches of BCI Technology

**Figure 2:**
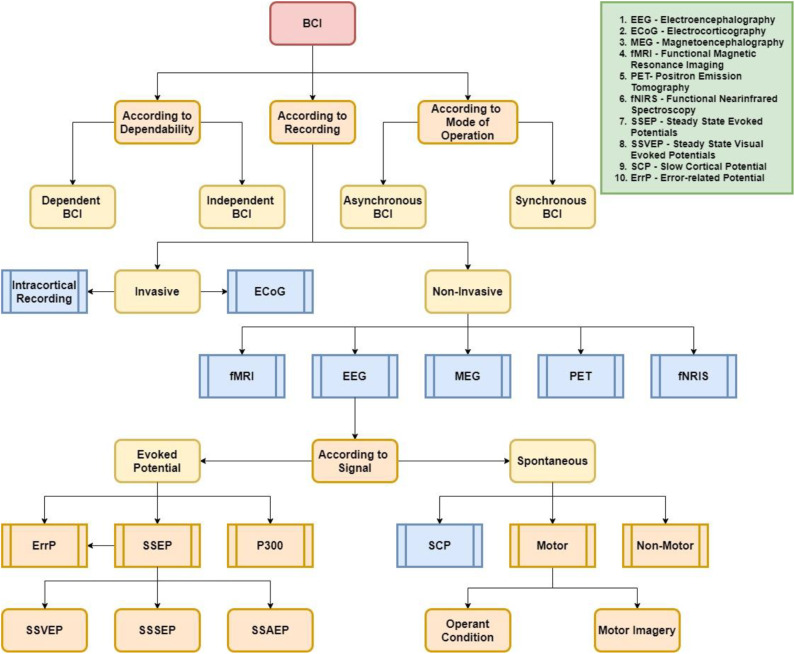
Classification of BCI systems in terms of dependability, recording method, and mode of operation.

Generally, BCI frameworks may be separated into a number of classes. Figure 2 illustrates the three categorization schemes, namely by dependability, recording technique, and method of operation (Lotte et al., 2015). Regarding dependability, BCI can be classed as either dependent or independent BCI. Dependent BCIs require some form of motor control by the user or healthy subjects, for instance, gaze control (Lalor et al., 2005). MI-based BCIs are an ideal example of dependent BCI systems and have been extensively utilized. Conversely, independent BCIs do not require any form of motor control by the user; this type of BCI is ideal for stroke patients or severely impaired patients. In Tello et al. (2016), an SSVEP-based independent BCI system was proposed to identify two different targets, and it was demonstrated to be successful.

With regard to recording method, BCI can be categorized into invasive and non-invasive. Microelectrode arrays are often required to be implanted inside the skull for invasive BCIs. Two common invasive modalities that have been reported in BCI research are intracortical recording and electrocorticography (ECoG). Conversely, if the brain signals are acquired by means of sensors placed on the scalp, it is known as non-invasive BCI. Amongst the non-invasive modalities often utilized are EEG, MEG, PET, fMRI, and fNIRS. In BCIs, EEG is the most widely employed non-invasive modality, where a variety of control signals, including SCP, SSVEP, MI, ErrP, and P300, can be evoked.

Finally, BCI can have either a synchronous or asynchronous mode of operation. The interaction between the user and the system may be either time-dependent or time-independent. In the event that the interaction is carried out within a certain period of time upon a cue imposed by the system, then the system is known as synchronous BCI. In contrast, in asynchronous BCI, the subject can generate a mental task at any period of time to interact with the application. In comparison with asynchronous BCI, synchronous BCIs are not user-friendly, but designing such a system is much easier than for asynchronous BCI (Bashashati et al., 2007b).

### Brain Activity Measurement Modalities

To avoid the risk of surgery, most BCI researchers prefer the non-invasive approach. EEG, MEG, PET, fMRI, and fNIRS are among the non-invasive modalities that are often utilized. The selection of the measurement method depends on a variety of parameters, for instance, spatial resolution, temporal resolution, invasiveness, measured activity, cost, and portability, amongst others. Owing to its desirable traits, namely high temporal resolution, low cost, ease of portability, and non-invasiveness, EEG is the most commonly employed neuroimaging modality among BCI researchers.

EEG records voltage fluctuations due to the flow of ionic current during synaptic excitations in the neurons of the brain (Baillet et al., 2001). In this modality, electrodes are attached to the scalp to obtain brain signals. Its non-invasive and inexpensive characteristics have made EEG the most popular modality among the BCI research community. The electrode number varies from 1 to 256 for different EEG headsets. The measured EEG signal is the voltage difference between the active and reference electrode over time, with its amplitude in micro-volts (μ*V*). Generally, the EEG amplitude ranges from −100 to +100 microvolts. The EEG signals can be categorized according to frequency bands, and each of these bands has specific biological significance. The EEG frequency bands with relevant characteristics are listed in Table 1 (Wang et al., 2016).

**Table 1:** EEG frequency bands with properties.

**Band**	**Frequency (Hz)**	**Amplitude (μV)**	**Location**	**Activity**
Delta	0.5–4 Hz	100–200	Frontal	Deep sleep
Theta	4–8 Hz	5–10	Various	Drowsiness, light sleep
Alpha	8–13 Hz	20–80	Posterior region of head	Relaxed
Beta	13–30 Hz	1–5	Left and right side, symmetrical distribution, most evident frontally	Active thinking, alert
Gamma	>30 Hz	0.5–2	Somatosensory cortex	Hyperactivity

### EEG Control Signals Used in BCI Applications

Some neurophysiological EEG signals have been decoded to enable the BCI to understand the subject's intentions, and these signals are known as EEG control signals. BCI aims to identify the specific neurophysiological signals of a given subject in order to associate a command to each of these signals. Some of these control signals are relatively easy to identify, as well as being relatively easy to control by the user. The extensively utilized EEG control signals include SCP, P300, MI, MRCP, ErrP, SSVEP, SSAEP, and SSSEP.

The movement-related cortical potential (MRCP) is a low-frequency negative shift in the EEG recording that takes place ~2 s prior to the production of voluntary movement. MRCP replicates the cortical processes employed in the planning and preparation of the movement (Shakeel et al., 2015). It is mainly beneficial for those BCI applications where the delay between the intention to act and the feedback from the system is crucial to induce plasticity.

The error-related potential (ErrP) has recently been utilized as an ERP component that can be used to correct BCI errors. The ErrP occurs when there is a mismatch between a subject's intention to perform a given task and the response provided by the BCI (Abiri et al., 2019). For instance, if a user wishes to move a cursor from the middle of a monitor to the left side of the monitor but the cursor erroneously moves to the right, an error-related potential will be generated. The ErrP is most pronounced at the frontal and central lobes. The delay and non-stationarity characteristics of this signal are still a challenge for real-time BCI implementation (Abiri et al., 2019).

Spontaneous signals are generated voluntarily by the user, without external stimulation, following an internal cognitive process. The most typical spontaneous signals used are undoubtedly sensorimotor rhythms. However, other neurophysiological signals have been used, such as slow cortical potentials or non-motor cognitive signals.

Slow Cortical Potentials (SCP) are very slow variations in cortical activity that can last from hundreds of milliseconds (ms) to several seconds (s) (Kleber and Birbaumer, 2005). It is possible to make these variations positive or negative via operant conditioning. As the control of SCP is achieved by operant conditioning, mastering such a signal generally requires a very long training time. This training by operant conditioning is even longer for SCP than for motor rhythms (Birbaumer, 2006). However, it seems that SCP would be a more stable signal.

Non-motor cognitive processing tasks are also extensively used to operate a BCI. These tasks are, for instance, mental mathematical computations, mental rotation of geometric figures, visual counting, mental generation of words, and music imagination, amongst others (Chiappa and Bengio, 2004). All of these mental tasks generate specific EEG signal variations in specific cortical regions and frequency bands, which make them relatively easy to identify.

#### Steady-State Evoked Potentials (SSEP)

SSEP appears when the user perceives a periodic stimulus like a flickering photo or an amplitude-modulated sound. An important characteristic of SSEP is that the stimulation frequency or harmonics is equivalent to the EEG signal frequencies (Gouy-Pailler et al., 2007). The stimulation of a fixed frequency evokes SSEP by yielding EEG activity of the identical frequency as the stimulation is generated (Maye et al., 2011). According to visual, auditory, and somatosensory stimulation, SSEP can be further divided into Steady-State Visually Evoked Potentials (SSVEP) (Valbuena et al., 2010), Steady-State Auditory Evoked Potentials (SSAEP) (Fairclough and Gilleade, 2014), and Steady-State Somatosensory Evoked Potentials (SSSEP) (Muller-Putz et al., 2006).

Every SSVEP-based BCI needs a specific number of visual stimuli that indicate specific BCI output commands. These stimuli flicker continuously, with distinguishable frequency bands ranging from 6 to 30 Hz. In the event that a subject concentrates on a particular flickering stimulus, an SSVEP with an identical frequency to that of the target flicker is generated. For example, if the frequency of the targeted stimulus is 15 Hz, the frequency of the generated SSVEP will also be 15 Hz. Therefore, the user pays attention visually to a target, and the BCI determines the target through analyzing the SSVEP features.

SSAEP are commonly extracted by trains of click stimuli, tone pulses, or amplitude-modulated tones, with a repetition or modulation rate between 20 and 100 Hz. The resulting brain response can be localized at the primary auditory cortex (Hill and Schölkopf, 2012). Although the SSAEP-based BCI system yielded promising results, only highly experienced users could maintain the high level of attention needed in order to obtain high accuracy (Punsawad and Wongsawat, 2017).

In the SSSEP paradigm, vibrotactile sensors are placed at pre-determined parts of the body, and these sensors generate stimulations at different frequencies (Hamada et al., 2014). The stimulations of these sensors will then be reflected in EEG signals recorded from the scalp. In comparison to visual- or sensorimotor rhythm-based BCI research, limited studies of SSSEP have been published. This is primarily due to the lack of a well-designed standard tactile stimulator with which to extract the SSSEP signals.

#### P300

BCI systems with P300 rely upon stimuli that flash in succession. These stimuli may be symbols or letters and are used for different BCI applications, for instance, controlling a robot arm, cursor, or mobile robot. P300 is generated in the Pz areas of the brain, ~300 ms after the stimulus is presented (Farwell and Donchin, 1988). It has been reported in the literature that the response's peak amplitude is much larger, even with less probable stimulus (John et al., 1996). Amongst the advantageous features of P300-based BCIs is that they do not require any form of training. However, it is worth mentioning that in the event that infrequent stimulus decreases the amplitude of P300 that, in turn, reduces the overall performance of the system.

#### Motor Imagery

Moving a limb or even contracting a single muscle changes brain activity in the cortex. Preparation for the movement or imagining movement [also known as motor imagery (MI)] generates oscillations in the brain motor areas known as sensorimotor rhythms (SMR). Increase and decrease of oscillatory activity in a particular frequency band are referred to as event-related synchronization (ERS) and event-related desynchronization (ERD), respectively. The most influential frequency bands for motor imagery are the alpha and beta brain waves. Activity invoked by the left and right hand MI is generated from the C3 and C4 areas of the brain, respectively, whereas the foot movement imagery is originated from Cz. Left and right foot movements are almost impossible to distinguish in EEG due to the fact that the corresponding cortical regions are extremely close. The cortical areas must be large enough to produce detectable patterns in the background EEG. The cortical areas of the left hand, right hand, tongue, and foot, are large and distinguishable. Thus, the movement of those body limbs via imagination can be controlled by BCI applications (Schlögl et al., 2005).

### EEG Acquisition Framework for BCI Application

The human brain consists of two main parts, i.e., the cerebral cortex and subcortical regions. The essential and vital functions, including body temperature, respiration, heart rate, and emotional responses, including reflexes, fear, learning, and memory, are controlled by the subcortical regions. Conversely, the cerebral cortex, commonly known as the cerebrum, regulates sensory and motor processing as well as higher-level functions, for example, language processing, pattern recognition, reasoning, and planning. The cerebral cortex is partitioned into two hemispheres, in which every hemisphere is classified into four lobes, namely the parietal, occipital, frontal, and temporal lobes. The parietal lobe is in charge of numerous functions, for instance, spelling, objects, manipulation, perception, and spatial awareness. Conversely, the language, memory, recognizing faces, and generating emotions are the main functions of the temporal lobe. The third lobe, i.e., the frontal lobe, involves organizing, social skills, planning, flexible thinking, problem-solving, conscious movement, attention, and emotional and behavioral control. The occipital lobe is related to interpreting visual stimuli. Additionally, another essential system of the human body is the nervous system, which is classified into two main parts: the central and peripheral systems. The spinal cord and the brain are the two parts of the central nervous system. In contrast, the peripheral nervous system includes the autonomic nervous system, which controls functions such as digestion, secretion of hormones, breathing, and heart rate.

**Figure 3:**
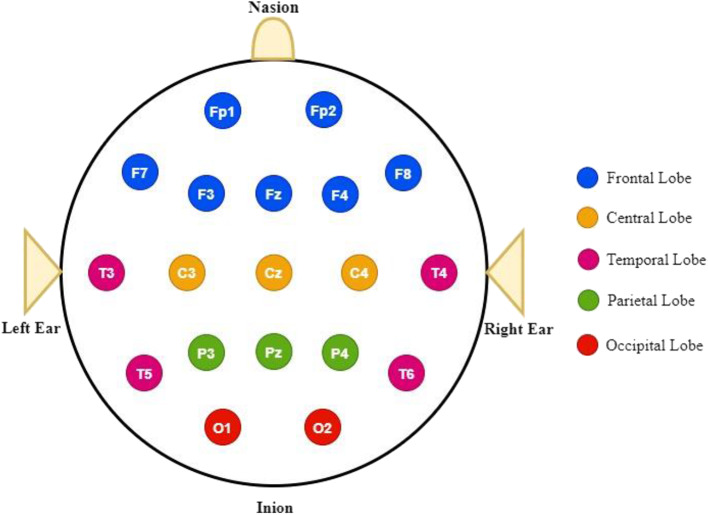
Standardized electrode placement scheme.

The 10/20 system is a universally recognized method that indicates the locations of electrodes on the scalp. The system depends on the connection between the electrode location and the underlying area of the cerebral cortex. The numbers 10 and 20 indicate that the distances among adjacent electrodes are either 10 or 20% of the total front-back or right-left distance of the skull. In each site, a letter is used to denote the lobe, whereas the hemisphere location is represented by a number. In the 10/20 system, the frontal, parietal, temporal, and occipital lobes can be denoted by the letters F, P, T, and O, respectively, as depicted in Figure 3. The central lobe is not included; the letter C is utilized only for identification purposes. Z (zero) implies that an electrode is placed on the midline. Even numbers (2, 4, 6, 8) are utilized to indicate the right hemispheres electrode positions, whereas left-hemisphere electrode positions are denoted by odd numbers (1, 3, 5, 7) (Rojas et al., 2018).

#### Hardware Technology for EEG Signal Acquisition

There are two main methods of acquiring EEG signals: wireless or wired. Typically, EEG signal measurements are performed using a number of electrodes varying from 1 to about 256. These electrodes are generally attached using an elastic cap. The contact between the electrodes and the skin is commonly enhanced through the utilization of a conductive gel or paste. However, this makes the electrode embedding procedure a generally tedious and lengthy operation. Nonetheless, it is worth noting that the use of dry electrodes, which do not require conductive gels or pastes, has been proposed and validated (Popescu et al., 2007). In spite of this success, it is worth pointing out that the performance of this method in terms of maximum information rate is, on average, 30% lower than that obtained with a BCI based on electrodes that employ conductive gels or pastes. Though the wired system is well-established, it has some notable limitations. It is evident that connection using wires between the electrodes and the acquisition part is often complicated, as it is a rather time-consuming procedure. Moreover, the user's movement is restricted owing to the tethered nature of cable constraints. Hence, wireless BCI systems have gained due attention, primarily owing to their ability to mitigate the aforesaid restrictions. One of the attractive natures of the wireless EEG headset is that it is non-invasive. Moreover, it does not hinder the motion of the user. Table 2 lists the types of EEG devices that have been reported in the literature with their specifications. It is evident that the selection of the type of EEG headset or device is dependent on the BCI application itself^1^.

**Table 2:** Summary table of recent EEG devices.

**Device name**	**No. of channels**	**Sampling frequency**	**Communication**	**No. of publications**
NeuroScan	SynAmps:64 Grael:32 NuAmps:40 Siesta:32	SynAmps:20 kHz Grael:4,096 Hz NuAmps:1,000 Hz Siesta:1,024 Hz	Wired	12,300
Brain Products	LiveAmp: 8/16/32	Between 250, 500, and 1,000 Hz	Wireless	6,690
BioSemi	16, 32, or 64	2/4/8/16 KHz	Wired	5,750
Emotiv	INSIGHT: 5 EPOC+: 14 EPOC FLEX: 32	128 Hz	Wireless	3,990
NeuroSky	1	512 Hz	Wireless	2,290
Advanced brain monitoring	ABM B-Alert X24: 24	256 Hz	Wireless	790
g.tec nautilus	64	500 Hz	Wireless	430
AntNeuro eego	64	2,048 Hz	Wireless	340
Neuroelectrics Enobio 32	32	500 Hz	Wireless	317
Muse	4	256 Hz	Wireless	207
OpenBCI	Up to 16 channels	256 Hz	Wireless	201
Cognionics Mobile	72	500–1,000 Hz	Wireless	128
mBrainTrain	24	250–500 Hz	Wireless	38
MyndBand EEG headset	3	512 Hz	Wireless	
Enobio	8, 20, or 32	500 Hz	Wireless	

### EEG Data Pre-processing Strategies

A small SNR and different noise sources are amongst the greatest challenges in EEG-based BCI application studies. Unwanted signals contained in the main signal can be termed noise, artifacts, or interference. There are two sources of EEG artifacts: external or environmental source and physiological source. The external sources of noise include AC power lines, lighting, and a large array of electronic equipment (from computers, displays, and TVs to wireless routers, notebooks, and mobile phones, amongst others). Physiological noise arises from an assortment of body activities due to movement, other bioelectrical potentials, or skin resistance fluctuations. The predominant physiological noises include electrooculographic activity (EOG, eye), electrocardiographic activity (ECG, heart), scalp-recorded electromyographic activity (EMG, muscle), ballistocardiographic activity (heart-related pulsatile motion), and respiration (Somers et al., 2018).

Pre-processing is a non-trivial process, as it is carried out to remove any unwanted components embedded within the EEG signal. Good preprocessing leads to an increase in the signal quality, which in turn results in better feature separability and classification performance. Simple low, high, and band-pass filters are the primary attempts to attenuate artifacts in the measured EEG. However, these are only effective when the frequency bands of the signal do not overlap (Sweeney et al., 2012). In case of spectral overlap, where artifacts are recorded with the EEG, alternative artifact removal techniques are required such as adaptive filtering, Wiener filtering, Bayes filtering (Sweeney et al., 2012), surface Laplacian transforms (Fitzgibbon et al., 2013), regression (Gratton et al., 1983), Common Average Referencing (CAR) (Zaizu Ilyas et al., 2015), EOG correction (Croft and Barry, 2000), and blind source separation (BSS) (Oosugi et al., 2017), as well as more modern attempts, for instance, the wavelet transform (WT) method (Punsawad and Wongsawat, 2017), empirical mode decomposition (EMD) (Zhang et al., 2008), Canonical Correlation Analysis (CCA) (de Clercq et al., 2006), and non-linear mode decomposition (NMD) (Iatsenko et al., 2015). It is worth noting that the BSS methods are also called component-based techniques, as they employ principal component analysis (PCA) or independent component analysis (ICA). Kilicarslan et al. (1976) proposed a quick artifact segment identification technique through the combination of dynamic time warping (DTW) and temporal motifs. Chavez et al. (2018) proposed a data-driven algorithm, namely surrogate-based artifact removal (SuBAR), to remove muscular and ocular artifacts effectively from EEG. A joint approach combining BSS and REG, the online EEG artifact attenuation technique, has also been proposed for BCI applications (Guarnieri et al., 2018). Although there is no single gold standard in EEG artifact removal algorithms, the authors of Urigüen and Garcia-Zapirain (2015) recommend using an ICA algorithm based upon second-order blind identification (SOBI) due to its success in removing different types of EEG contaminants.

Real-time BCI applications require artifact removal methods that are automatic and of low computational cost. Regression and filtering approaches can be executed automatically when they have a reference signal. Moreover, BSS methods will be automatic when there is a subsequent procedure. Although ICA is the most commonly used technique among these BSS methods, it disregards the temporal or spatial relations within sources, which will result in the loss of relevant information. Nevertheless, a CCA algorithm can mitigate this problem as it takes little computational time, which makes the algorithm applicable for real-time performance. Another factor that should be taken into account is the number of measurement channels. It should be noted that for home healthcare environments, fewer channels are often expected. BBS algorithms cannot be utilized in such a situation, due to the principle of BSS that it requires more channels in order to allow it to be more accurate. However, it should be noted that wavelet transform and EMD-based methods can be executed with a single channel, as they can decompose from a single record into multiple components (Chen et al., 2014). However, a reduction in the number of measurement channels will cause an increase in computational complexity, which will not be suitable for BCI applications.

In addition, it is worth noting that automatic methods are not commonly used for artifact removal, as there are multiple types of artifacts that exist in the recordings. Hence, the availability of reference signals will improve the accuracy and robustness of artifact removal by providing satisfactory complementary information. Also, the information on the artifact epochs obtained by the reference channel will reduce the computational cost. However, having a reference channel for each muscle contributing to EEG muscle artifacts is not feasible. Apart from the aforementioned methods, there are plenty of innovative and efficient approaches for artifact removal that have been recently proposed. One recently emerging BSS algorithm, independent vector analysis (IVA), integrates the advantages of CCA and ICA into one single framework. This technique could remove muscle artifacts by synchronously extracting the sources with maximal independence and maximal autocorrelation (Chen et al., 2017a,b). In addition, the combination of EEMD and IVA has been demonstrated to outperform other existing methods in a situation where there are few channels (Xu X. et al., 2018). More recently, a modified joint BSS approach and quadrature regression IVA (q-IVA) provided a more effective artifact removal technique in both the time and frequency domains, paving the way for future research (Lee S. et al., 2019). Dhindsa (2017) proposed a filter-bank-based supervised machine learning approach to detect artifacts from a single channel, and the approach outperformed statistical thresholding for EEG artifact rejection due to its ability to identify small artifacts in the presence of high-amplitude EEG. Mohammadpour and Rahmani (2017) have utilized an HMM architecture to remove eyeblink artifacts. Contrary to conventional algorithms, machine learning-based approaches have gained due attention, particularly for their ability to identify artifacts. To attenuate eye blink artifacts, a multichannel Weighted Weiner filter has been proposed (Manojprabu and Sarma Dhulipala, 2020), where Hierarchical Fully Connected Topology (HFCT) and *Ad-hoc* Nearest-Neighbor Topology (ANNT) are utilized. The proposed approach provides 5% better results for artifact attenuation when compared with the other existing approaches like PCA and ICA. However, it should be noted that the proposed approach has not been employed in real medical devices.

### Feature Extraction Approaches in EEG-Based BCI Systems

After the noise removal phase, the most discriminative and non-redundant information within the EEG is extracted through different feature extraction techniques. Time-domain, frequency-domain, time-frequency domain, and spatial domain are the popular types of feature extraction techniques in EEG-based BCIs.

A typical time-domain-based feature extraction approach, autoregressive (AR) modeling, is a linear regression of the current observation of the series against one or more earlier observations. A combination strategy of feature extraction, where each feature vector consists of AR coefficients and approximate entropies, was also proposed. In many recent articles (Lawhern et al., 2012; Zhang and Xiaomin, 2015; Chai et al., 2017b), the AR model has been implemented as the strategy of feature extraction in EEG-based BCI systems. AR models are preferred by researchers due to their resolution, smoother spectra, and applicability to short segments of data. Lower model orders represent the signal poorly, while higher orders increase noise. Hence, identifying the appropriate AR modeling order is an open challenge. Conventional ways of modeling order estimation incorporate a Bayesian information criterion, Final prediction error, or Akaike Information Criterion (AIC). It was hypothesized in Atyabi et al. (2016) that an adequate mixture of AR features derived from various AR modeling orders is a better representative of the underlying signal compared with any fixed modeling order. For the detection of drowsiness state from EEG signals, the analysis of respiratory rate variability from EEG (Guede-Fernández et al., 2019), adaptive Hermite decomposition (Taran and Bajaj, 2018), and RR time series (Tripathy and Rajendra Acharya, 2018) have been employed to extract features. In emotion recognition using an EEG signal, the fractal dimension of raw signals has been implemented to extract the feature by using the Higuchi technique (Anh et al., 2012; Kaur et al., 2018). In AydIn et al. (2009), the authors proposed the use of log energy entropy to extract EEG features; this approach could investigate how much randomness is captured in the signal. A hybrid feature extraction technique consists of PCA, and the cross-covariance technique has been developed in Zarei et al. (2017) to excerpt discriminatory information from the mental states of EEG.

Frequency-domain analysis has also been employed to extract features from different EEG-based BCIs. Among frequency-domain-based techniques, there are those that use fast Fourier transform (FFT) (Hortal et al., 2015; Djamal et al., 2017; Bousseta et al., 2018; Yang C. et al., 2018), power spectral density (PSD) (Chiappa and Bengio, 2004; Carlson and Millan, 2013; Mara et al., 2013; Pham et al., 2013; Bascil et al., 2016; Liu Y. et al., 2017; Nguyen et al., 2017; Chakladar and Chakraborty, 2018b), band power (Mandel et al., 2009; Serdar Bascil et al., 2015; Kreilinger et al., 2016), and spectral centroid (Murugappan et al., 2014). The PSD of a signal can be calculated through the FFT and Welch's method (Oikonomou et al., 2017). Welch's method reduces the artifacts in the PSD, in contrast to FFT, but produces a poorer frequency resolution. Another frequency domain-based feature extraction technique that does not require FFT to compute the PSD is local characteristic-scale decomposition (Liu A. et al., 2017). This procedure disintegrates the raw data into inherent segments that convey the properties of the primary signal. Fourier analysis decomposes the signal into its frequency components and determines their relative strengths. Due to the non-stationarity and non-Gaussianity properties of the EEG signals, classic spectral analysis techniques are not suitable for extracting useful and important information. Gursel Ozmen et al. (2018) introduced a biologically inspired frequency domain-based feature extraction approach that extracts the most discriminative spectral features from the PSD of the EEG signals. Meziani et al. (2019) presented novel spectral estimators, namely the quantile periodogram, and the lasso quantile periodogram, which are based on quantile regression and L1-norm regularization, respectively.

The use of spectral characteristics for feature extraction is sometimes ineffective due to the absence of temporal characteristics. Similarly, time-domain interpretation occasionally neglects spectral characteristics that may be important to the classifier. To overcome the shortcomings of a single domain that is either time domain or frequency domain, time-frequency analysis is assumed to be able to mitigate the issue as it leverages both domains. This approach could be promising for EEG-based BCIs. A variety of time-frequency-based feature extraction approaches have been employed in EEG-based BCIs. The most widespread approaches are short-time Fourier transform (STFT) (Tabar and Halici, 2017; Chaudhary et al., 2019; Ha and Jeong, 2019; Tian and Liu, 2019), continuous wavelet transform (CWT) (Borisoff et al., 2004; Lee and Choi, 2019; Ieracitano et al., 2020), discrete wavelet transform (DWT) (Guo et al., 2015; Bajwa and Dantu, 2016; Djamal and Lodaya, 2017; Ji et al., 2019; Lin and She, 2020), and wavelet packet decomposition (WPD) (Bong et al., 2017; Dhiman et al., 2018; Wang et al., 2019). CWT (Ortiz-Echeverri et al., 2019; Mammone et al., 2020), and STFT (Dai et al., 2019) have been utilized to generate spectral images that can be classified through deep learning approaches. An EEG-based motor planning exercise was investigated by Mammone et al. (2020), where a time-frequency map, generated through beamforming and CWT, was utilized as input to the CNN. Decomposition techniques, for instance, DWT and WPD, are efficacious because significant information is carried in different EEG bands (Kevric and Subasi, 2017), and these approaches are capable of decomposing the brain waves at multiresolution and multiscale (Li et al., 2016a). Moreover, they are able to extract dynamic features, which is crucial for EEG signals due to their non-stationary and non-linear characteristics (Kevric and Subasi, 2017). In Kevric and Subasi (2017), three distinct decomposition techniques, namely, WPD, EMD, and DWT, have been investigated to gain optimum accuracy. Higher-order statistics (HOS) features have been extracted from the decomposed EEG sub-bands. The frequency resolution of DWT coefficients is comparatively lower than that of WPD, and the deficiencies of wavelet strategies could be neutralized by HOS.

Zhou et al. (2018) combined the utilization of DWT and Hilbert transform (HT) for feature extraction. The EEG signal is decomposed through DWT, and the wavelet envelope of the decomposed sub-bands was computed through HT. They utilized both time-series and envelope information, which assisted in obtaining optimum accuracy. Göksu (2018) proposed wavelet packet analysis (WPA) to extract features from an EEG-SCP response. The WPA sub-images were further studied through log energy entropy. Yang et al. (2016), proposed Fisher wavelet packet decomposition (WPD)-CSP for extracting features, in which EEG channels are decomposed by WPA, the average power of each subband is calculated, and then, finally, CSP is employed to the selected subbands.

Another powerful feature extraction approach known as the common spatial pattern is extensively utilized in EEG-based BCI (Zhang R. et al., 2019). This technique utilizes a spatial filtering method that converts brain waves into a unique space where the variance of one group is magnified, while lower variance is seen in the remaining group. The pure CSP approach sometimes cannot achieve sufficient performance due to the subject-specific optimal frequency band. Hence, the choice of an optimized filter band may enhance performance, but the selection of the optimal sub-band through pure CSP takes a large amount of time. To overcome this issue, numerous changes have been applied to the CSP. The common spatio-spectral pattern approach (CSSP) combines an FIR filter with a CSP algorithm and was observed to improve performance relative to pure CSP (Reddy et al., 2019). Common sparse spatio-spectral patterns (CSSSP) (Dornhege et al., 2006) are a comparatively more advanced procedure where the common spectral patterns across channels are investigated. In sub-band common spatial pattern (SBCSP) (Khan et al., 2019), EEG is first filtered at different sub-bands, and then CSP features are calculated for each of the bands. Frequency bands of the CSP, for instance, filter bank CSP (FBCSP), were implemented in Korik et al. (2019), whereas wavelet CSP (WCSP) has been implemented (Lin et al., 2019) by considering the effect of frequency resolutions. However, these strategies are not significantly relevant to EEG data from selected electrodes. To mitigate this issue, a new technique for feature extraction from selected channels known as regularized CSP (RCSP) has been proposed (Jin et al., 2019).

The efficient frequency recognition algorithm in SSVEP-based BCIs performs a crucial role in overall system performance. Among these algorithms, the most prevalent are based on multivariate statistical algorithms, for instance, canonical correlation analysis (CCA) (Chen et al., 2015) and multivariate synchronization index (MSI) (Zhang Y. et al., 2016). Recently, another MSI-based frequency recognition approach known as CORRCA, which is based on correlated component analysis (COCA), has been proposed (Zhang et al., 2018). The CORRCA approach performs substantially better than the state-of-art CCA process. Authors in Zhang Y. et al. (2019) proposed a hierarchical feature fusion architecture that consists of the spatial dimension and frequency dimension to improve the performance of frequency identification techniques in SSVEP-based BCI.

Batres-Mendoza et al. (2016) proposed a novel feature extraction approach based on quaternions, which represent objects within a three-dimensional space with regards to their orientation and rotation. Islam et al. (2018) proposed a multiband tangent space mapping with sub-band selection (MTSMS) approach to improve EEG-MI classification accuracy, and the authors claim that the proposed framework outperforms state-of-the-art methods. Authors in Lee S. B. et al. (2019) investigated the comparative analysis of EEG features in three different domains, namely spectral, temporal, and spatial, to classify multi-class MI data. According to their investigation, the time-domain parameter (TDP) has been observed to be superior as compared to the CSP and PSD. Another study explored the use of tunable Q-factor wavelet transform (TQWT) for the identification of drowsiness EEG signals (Al Ghayab et al., 2019). With this approach, TQWT decomposes the EEG signals into band-limited sub-bands, and the drowsiness and alertness EEG signal characteristics from TQWT-provided sub-bands are extracted using time-domain measures. These measures are based on the statistics of Hjorth mobility. Moreover, a novel hybrid feature extraction technique has been proposed (Asadur Rahman et al., 2019), which consists of PCA and t-statistics. In Guede-Fernández et al. (2019), the analysis of respiratory rate variability of EEG has been utilized to monitor the state of drowsiness in a driver.

### Classification Methods

To operate a BCI system, the subject needs to create various brain activity patterns that can be identified by the system and translated into commands. It is worth noting that either regression or classification algorithms could be utilized to achieve the said objective. However, the usage of classification algorithms is presently reported to be the most popular approach (Lotte et al., 2007). The design of the classification step includes the choice of one or several classification algorithms from many alternatives. Numerous classification algorithms have been presented in the published EEG-based BCI literature, for instance, support vector machine (SVM), neural network (NN), linear discriminant analysis (LDA), Bayesian classifier, k-nearest neighbor (*k*-NN), as well as deep learning and its iterations. The aforesaid classifiers are described briefly, and their essential properties for BCI applications are highlighted.

#### Conventional Machine Learning Approaches in EEG-Based BCIs

The *k*-NN algorithm depends on the principle that the features corresponding to the several classes will form individual clusters in feature space. The features that are closer to each other are recognized as neighbors and are therefore grouped together. This classifier takes k metric distances into account between the test sample features and those of the nearest classes in order to classify a test feature vector. The metric distances are a measure of the similarities between the features of the test vector and the features of each class (Nicolas-Alonso and Gomez-Gil, 2012). It is worth highlighting that the *k*-NN algorithms are not exceptionally popular in the BCI community because they are known to be very sensitive to the dimensionality of the feature vector (Borisoff et al., 2004). Nonetheless, when the algorithm is utilized in BCI systems with low-dimensional feature vectors, the algorithm could be useful. Thus, the *k*-NN algorithm can provide good results when it is combined with other efficient feature selection and reduction algorithms. In *k*-NN architecture, the number of neighbors and the type of distance metrics are the key factors.

LDA is employed to find the linear combinations of feature vectors that describe the characteristics of the corresponding signal. The LDA seeks to separate two or more classes of objects or events representing different classes. It utilizes hyperplanes to accomplish this mission. The isolating hyperplane is achieved by searching for the projection that maximizes the distance among the classes' means and minimizes the interclass variance (Abdulkader et al., 2015). This technique has a very low computational requirement, and it is simple to use. The LDA has been successfully applied in a variety of BCI systems, for example, motor imagery-based BCI, P300 speller, multiclass, or asynchronous BCI (Long et al., 2012a). However, while it provides expected outcomes due to its immunity to non-stationary issues, its linearity can cause performance degradation in a few circumstances containing complex non-linear EEG data. Moreover, numerous updated algorithms have been presented depending on LDA, for example, Fisher LDA (FLDA) as well as Bayesian LDA (BLDA) (Hoffmann et al., 2008). FLDA does not work well if the number of features becomes too large in relation to the number of training examples. This issue is called the small sample size problem (Hoffmann et al., 2008). On the other hand, the BLDA is considered as an expansion of FLDA that mitigates the small sample size problem through the incorporation of a statistical method called regularization. The regularization is estimated through Bayesian analysis of training data and is utilized to prevent the overfitting problem of high-dimensional as well as possibly noisy datasets. Overfitting means that the classifier has lost generality and is therefore undesirable in a classifier. If a classifier is overfitted, it is only able to classify the training data or similar data. Unlike FLDA, the BLDA algorithm gives higher classification accuracy and bitrates, particularly in those situations where the size of the training sample is large (Hoffmann et al., 2008). Furthermore, BLDA requires slightly more computational time, which is a crucial constraint in real BCI systems.

SVM is a classifier that builds a hyperplane or set of hyperplanes for separating the feature vectors into several classes. However, in contrast to LDA, SVM selects the hyperplanes that maximize the margins, that is, the distance between the nearest training samples and the hyperplanes (Burges, 1998). SVM that empowers classification by utilizing linear decision boundaries is called linear SVM. This type of classifier has been applied successfully to a moderately large number of synchronous BCI problems (Garrett et al., 2003; Rakotomamonjy et al., 2005). However, it is advantageous to make non-linear decision boundaries with a low increment of the classifier's complexity by utilizing the “kernel trick.” The kernel usually utilized for BCI research is the Gaussian or Radial Basis Function (RBF) kernel. The corresponding SVM is called Gaussian SVM or RBF SVM. The RBF SVM has also shown to be robust for achieving good results in BCI applications (Garrett et al., 2003; Kaper et al., 2004; Rakotomamonjy et al., 2005). Generally, the SVM has been broadly recognized as the simpler algorithm used in BCI applications. In addition, the algorithm is shown to be robust with a high-dimensional dataset, which implies that a large training set is not required for good outcomes, even with high-dimensional feature vectors (Kaper et al., 2004). It is worth noting that these favorable circumstances do not hinder the execution speed during the integration of real-time BCIs (Thulasidas et al., 2006).

The neural network (NN) has the special capacity to extract patterns and identify trends that seem to be complicated, either by humans or by computerized techniques. A trained NN system can be considered as an “expert” in the classification of information that it has been provided to analyze. This algorithm is one of the fundamental tools utilized in machine learning. The term “neural” denotes that it is considered to be a brain-inspired system that is intended to replicate the way that humans learn. A NN consists of input, output, and hidden layers. A hidden layer consists of units that transform the input into something that the output layer can utilize. It is an excellent tool for discovering patterns that are too complex or numerous for a human programmer to extract and to teach the machine to perceive. One of the most well-known ANN structures is the multilayer perceptron (MLP) introduced by Rumelhart et al. (1986). MLPs are flexible classifiers with the ability to classify any number of classes as well as to adapt to various different sets of problems. In BCIs, the MLP has been applied to classify two, three, and five tasks and to design synchronous (Haselsteiner and Pfurtscheller, 2000) as well as asynchronous (Millan and Mourino, 2003) BCIs. Additionally, the MLP has been utilized to preprocess EEG signals prior to the feature extraction step rather than the classification step to improve the separability of EEG features (Coyle et al., 2010). Other than MLP, numerous sorts of NN architecture have been utilized in the design of BCI systems, including Fuzzy ARTMAP Neural Networks, Finite Impulse Response Neural Networks (FIRNN) or Probability estimating Guarded Neural Classifiers (PeGNC), Probabilistic Neural Networks (PNN), Time-Delay Neural Networks (TDNN) or Gamma dynamic Neural Networks (GDNN), Learning Vector Quantization (LVQ) Neural Networks, Bayesian Logistic Regression Neural Networks (BLRNN), RBF Neural Networks, and Adaptive Logic Networks (ALN), amongst others.

A Hidden Markov Model (HMM) is a Bayesian classifier that produces non-linear decision boundaries. An HMM is a sort of probabilistic automaton that gives the likelihood of observing a given sequence of feature vectors (Rabiner, 1989). For BCI, generally, these probabilities are Gaussian Mixture Models (GMM) (Obermaier et al., 2001). HMMs are perfectly appropriate algorithms for the classification of time series (Rabiner, 1989). As the EEG components that are used to operate BCI have specific time courses, HMM is applicable to the classification of temporal sequences of BCI features (Obermaier et al., 2001; Cincotti et al., 2003), even for classifying raw EEG signals.

#### Deep Learning Approaches in EEG-Based BCIs

The ability to acquire a robust automatic classification of EEG signals is an essential step toward making the use of EEG more practical in many applications and less reliant on trained professionals (Alexander et al., 2018). It is worth noting that although conventional BCI systems have made tremendous advances in the past few decades, nonetheless, the research still faces significant challenges in EEG classification. The challenges include various biological and environmental artifacts in EEG, a low SNR, and dependency on human expertise for extracting meaningful features. In addition, most existing machine learning research, if not all, centers on static data and, hence, is not able to classify rapidly changing brain signals accurately (Lotte et al., 2018). Of late, the availability of large EEG data sets has led to the utilization of Deep Learning (DL) architectures, especially to uncover relevant information from the signals that were not possible to acquire via conventional approaches and has shown success in addressing the aforesaid challenges. Fundamentally, DL is a specific machine learning algorithm in which the features and the classifier are jointly learned directly from data (Zhang X. et al., 2019). DL algorithms have been explored for almost all major types of EEG-based BCI systems, namely P300, SSVEP, motor imagery (MI), SCP, and passive BCI (for emotions and workload detection). Here, a number of prevalent DL models including convolutional neural networks (CNN), deep belief networks (DBN) restricted Boltzmann machines (RBM), recurrent neural networks (RNN), a stacked autoencoder (SAE), and generative adversarial networks (GAN) will be discussed briefly with regards to their employment in BCI research.

A Convolutional Neural Network (CNN) is a special type of neural network architecture that is specialized in spatial information exploration. CNN contains at least one convolutional layer, and this layer maps input to an output through a convolution operator (Fan et al., 2019; Zhang X. et al., 2019). In BCI research, CNN is assumed to capture the distinctive dependencies amongst the patterns associated with different brain signals (Lotte et al., 2018). Recently, a considerable amount of studies (Tang et al., 2017; Aznan et al., 2018; Dose et al., 2018; El-Fiqi et al., 2018; Shojaedini et al., 2018; Wang et al., 2018; Waytowich et al., 2018; Amber et al., 2019; Amin et al., 2019; Nguyen and Chung, 2019; Olivas-Padilla and Chacon-Murguia, 2019; Tayeb et al., 2019; Xu et al., 2019) on the employment of CNN architecture in EEG-based BCI systems have been published. In Olivas-Padilla and Chacon-Murguia (2019), the classification of multiple MI using CNN was explored, with the features being extracted by a variety of Discriminative Filter Bank Common Spatial Patterns (DFBCSP). Conversely, the authors in Xu et al. (2019) presented a wavelet transform time-frequency image coupled with a CNN-based approach in classifying EEG MI, and a classification accuracy of 92.75% was attained. Tayeb et al. (2019) classified raw EEG MI signals using a CNN architecture, achieving an accuracy of 84%, and this model has been successfully adopted in a real-time robotic arm control system. A CNN-based multilevel feature fusion model was proposed in Amin et al. (2019) for motor imagery EEG classification. Three other studies also employed CNN for EEG MI classification with reported the classification accuracies of 80, 93, and 86%, respectively (Tang et al., 2017; Dose et al., 2018; Wang et al., 2018). It should also be noted that the CNN model has also been employed in SSVEP-based BCI systems. A novel CNN approach for the classification of raw SSVEP EEG signals was proposed in Aznan et al. (2018). Here, the CNN architecture achieved a classification accuracy of 96%, which is significantly better than other competing DL approaches. In El-Fiqi et al. (2018), raw SSVEPs were classified using CNN for person identification and verification. In addition, a 1-D CNN was employed for SSVEP frequency detection with an average accuracy of 97.4% (Nguyen and Chung, 2019). A compact-CNN approach was proposed in Waytowich et al. (2018), and it was able to decode signals from a 12-class SSVEP dataset with a mean accuracy of ~80%. With regard to the use of CNN on P300, Amber et al. (2019) presented a lie detection system from the P300 signals with an accuracy of 99.6%. In addition, a new adaptive hyperparameter-tuning method is proposed in Shojaedini et al. (2018) to improve the training of CNN in P300 signal detection. It was established from the study that the proposed method is able to improve the classification accuracy by 6.44% against the conventional Naive hyperparameter tuning method.

A deep belief network (DBN) is a probabilistic generative model consisting of a sequence of restricted Boltzmann machine (RBM) architectures (Abbas et al., 2019). The top two layers in DBN are connected without directions, while the lower layers are connected with directions. The RBM consists of a visible layer and a hidden layer, and the connection lines between these two layers are undirectional (Abbas et al., 2019). Several studies have explored MI classification with DBN (An et al., 2014; Tang et al., 2015; Lu et al., 2017; Ortega et al., 2017). In Lu et al. (2017), a novel deep learning scheme based on RBM was proposed for EEG MI classification in which FFT and wavelet package decomposition are obtained to train three RBMs. These RBMs are then stacked up with an extra output layer to form a four-layer frequential DBN. The authors of Tang et al. (2015) proposed an EEG MI data recognition technique using DBN. The findings from the study showed that the recognition rate of EEG MI data based on a DBN is better than that with the conventional SVM model. A novel technique of classification of imagined speech in EEG was proposed in Lee and Sim (2015), where the classification accuracy obtained was 87.96% with DBN. A P300-based Guilty Knowledge Test system was proposed in Kulasingham et al. (2016). Here, the DBN architecture was used to classify the P300 wave with an accuracy of 86.9%, and the input to this classifier was the filtered EEG signal without any feature extraction. Another P300 potential detection method based on DBN has been proposed in Lu et al. (2018), where the average accuracy attained was 84.3%. A DBN architecture has also been exploited successfully for EEG-based emotion recognition (Zheng and Lu, 2015; Huang et al., 2017). An EEG-based emotion classification framework based on combining emotional patches and a DBN model was proposed in Huang et al. (2017), and it was reported that a classification accuracy of 94.92% was achieved, outperforming other traditional methods. The authors of Kawde and Verma (2017) implemented an effective recognition system to examine the emotional state of a human being based on DBN. The experiment was performed on a benchmark DEAP database, and the accuracies achieved were 78.28, 70.33, and 70.16% for valence, arousal, and dominance, respectively. In Bablani et al. (2018), a system for identifying deceit from EEG has also been proposed. A DBN was developed with four RBMs stacked together, and EEG data in the form of time-frequency was fed to this DBN. The accuracy of this system was recorded at 81%. In Chai et al. (2017a), an EEG-based driver fatigue classification between fatigue and alert states was investigated. The system employs an AR model as the feature extraction algorithm and a sparse-DBN as the classification algorithm. It was shown from the study that a classification accuracy of 93.1% was attained.

RNN architecture is a powerful deep learning classification method that is specifically applied to sequential data. This type of DL architecture is able to analyze the overall logical sequence between the input information. These logical sequences are rich in content and possess a complex time relationship with each other. The key concept of RNN is that the hidden state of the current network will retain the previous input information, and it is used for the next current network (Li et al., 2019). There are two typical RNN architectures that have attracted much attention and achieved great success: long short-term memory (LSTM) and gated recurrent units (GRU). Two notable studies have been carried out to recognize the EEG-based sleep stage using RNN architecture (Michielli et al., 2019; Wang and Wu, 2019; Zhang T. et al., 2019). A novel cascaded RNN architecture based on LSTM blocks was proposed in (Michielli et al., 2019) for the automated scoring of sleep stages using EEG, and an average classification accuracy of 86.7% was achieved. The authors of Wang and Wu (2019) also developed an automatic sleep stage classification system where they proposed an RNN based on the attention mechanism and bidirectional LSTM. This architecture provided better performance than the C-CNN model but requires more training time. A novel DL framework called spatial-temporal RNN (STRNN) was proposed in Zhang T. et al. (2019), where both spatial and temporal information were integrated for feature learning. The authors claimed that the experimental results based on STRNN were more competitive than the state-of-the-art methods for emotion recognition. In another study (Liu et al., 2018), the combination of temporal attention and band attention mechanisms based on multi-layer LSTM-RNN architecture was proposed for emotion recognition. Another study (Jawed et al., 2018) distinguished visual and non-visual learners by considering the wavelet features of EEG alpha and beta bands. The LSTM-based RNN framework was also used for classification purposes, and the mean training accuracy was 87.5 and 86% for beta and alpha bands, respectively. In relation to EEG MI classification, Ma et al. (2018) proposed a pure RNN-based parallel method for encoding spatial and temporal raw data with bidirectional LSTM and standard LSTM, respectively, reporting an average accuracy of 68.20%. A deep RNN with a sliding window cropping strategy (SWCS) to classify EEG MI signals was investigated in Luo et al. (2018). In addition, an LSTM-RNN architecture for an EEG MI classification model was proposed in Li et al. (2016b), where DWT was applied to extract the time-frequency features. A novel system for cross-day workload estimation using EEG has also been proposed by Hefron et al. (2017), where the authors applied an LSTM-based RNN architecture, and the average classification accuracy achieved was 93.0%.

An autoencoder (AE) is a DL approach used for unsupervised feature learning with efficient data encoding and decoding. In the encoding phase, the input samples are often mapped in the lower dimensional feature space with a constructive feature representation (Alom et al., 2019). This approach can be repeated until the desired feature dimensional space is reached. Conversely, in the decoding phase, actual features are regenerated from the lower-dimensional features with reverse processing (Alom et al., 2019). It should be pointed out that the target output of the autoencoder is the autoencoder input itself. There are a number of notable AE architectures, i.e., Stacked Autoencoder (SAE), Variational Autoencoder (VAE), and Generative Adversarial Networks (GAN), that have been employed in EEG signal processing investigations (Tsinalis et al., 2016; Vareka and Mautner, 2017; Yin and Zhang, 2017; Ditthapron et al., 2018; Idowu et al., 2018; Nair et al., 2018; Rundo et al., 2019). The authors of Rundo et al. (2019) developed a drowsiness detection system from EEG using stacked AE and achieved an accuracy of 100% in discriminating drowsy from wakeful. Idowu et al. (2018) proposed a DL-based classification of EEG signals for given visual stimuli by showing familiar and unfamiliar faces. The preprocessed signal was fed to an AE that yielded a mean accuracy of 82.21%. In Nair et al. (2018), five-class EEG MI data was classified, where SAE was applied to generate the features, and a softmax layer was then used for classification purposes. The proposed method produced an overall accuracy of 98.9%. In Ditthapron et al. (2018), the authors proposed a multitask autoencoder-based model known as the ERP encoder network (ERPENet) that can be applied to any ERP-related tasks. In Vareka and Mautner (2017), an SAE architecture was proposed for P300 wave detection, and the trained SAE achieved a classification accuracy of 69.2%. An automatic sleep stage scoring model that uses a single channel of EEG was proposed in Tsinalis et al. (2016). Here, the methodology is based on time-frequency analysis and stacked sparse autoencoders (SSAEs). The overall accuracy attained was 78%. With regard to the mental workload (MW) classification, several studies have been carried out (Yin and Zhang, 2017; Yang et al., 2019; Yin et al., 2019). An adaptive Stacked Denoising Auto Encoder (SDAE) was developed in Attia et al. (2018) to tackle cross-session MW classification from EEG, and it was reported that the proposed classifier achieved an accuracy of 95.5%.

Apart from the aforesaid standalone DL models, researchers have attempted to hybridize different DL models in EEG-based BCI investigations (Narejo et al., 2016; Attia et al., 2018; Yang J. et al., 2018; Dai et al., 2019; Kanjo et al., 2019), with encouraging classification accuracies. In Narejo et al. (2016), the authors developed a system for predicting eye state from EEG signals using a hybrid DL architecture consisting of DBN and SAE. The accuracy of this hybrid model was reported to be as high as 98.9%. Another hybrid DL architecture based on CNN-RNN was proposed in Attia et al. (2018) to classify SSVEP signals in the time domain directly, and it achieved an accuracy of 93.59%. Kanjo et al. (2019) proposed a hybrid approach that applied CNN and LSTM-RNN on the raw sensor data. Through this method, the need for manual feature extraction is eliminated. The results show that the adoption of DL approaches is effective in human emotion classification when a large number of sensor inputs are utilized (with an average classification accuracy of 95%). Dai et al. (2019) proposed a hybrid DL model where a CNN architecture was combined with a VAE for EEG MI classification. In addition, an LSTM-CNN-based hybrid model has also been proposed by Yang J. et al. (2018) for EEG MI classification.

The fundamental idea of a Riemannian geometry classifier (RGC) is to map the data directly onto a geometrical space equipped with a suitable metric (Lotte et al., 2018). In such a space, data can be easily manipulated for several purposes, such as averaging, smoothing, interpolating, extrapolating, and classifying. In the case of EEG data, the power and the spatial distribution of EEG sources can be considered fixed for a given mental state, and such information can be coded by a covariance matrix (Lotte et al., 2018). Riemannian geometry studies smooth curved spaces that can be locally and linearly approximated. The curved space is named manifold, and its linear approximation at each point is known as the tangent space (Lotte et al., 2018). Riemannian geometry has been successfully utilized in many BCI classification problems (Kalunga et al., 2016; Congedo et al., 2017; Wu et al., 2017; Yger et al., 2017; Gaur et al., 2018; Guan et al., 2019; Han et al., 2019; Majidov and Whangbo, 2019) and has demonstrated superior performance. In Han et al. (2019), the authors implemented an EEG-based endogenous BCI system for online binary communication by a completely paralyzed patient. An online classification accuracy of 87.5% was achieved when the Riemannian geometry-based classification was applied to real-time EEG data. A number of investigations (Gaur et al., 2018; Guan et al., 2019; Majidov and Whangbo, 2019) have employed Riemannian geometry for EEG MI classification purposes. The authors of Majidov and Whangbo (2019) proposed a Riemannian geometry-based architecture for EEG MI classification. They combined the PSD features with covariance matrices mapped onto the tangent space of a Riemannian manifold, and an average classification accuracy of 87.94% was obtained. The use of a Riemannian geometry framework for EEG MI classification has also been presented in Gaur et al. (2018), where the EEG signals were preprocessed using a subject-specific multivariate empirical mode decomposition (SS-MEMD)-based filtering method. They achieved a mean Kappa value of 0.60. Kalunga et al. (2016) investigated the efficiency of Riemannian geometry on SSVEP wave classification for a four-class BCI application. In the study, the minimum distance to Riemannian mean (MDRM) algorithm achieved an average classification accuracy of 90.47+/7.8% and an ITR of 16.37 ± 5.3 bits/min. A novel feature extraction approach based on Riemannian geometry was proposed in Wu et al. (2017), in which a spatial filter is first used to increase the EEG signal quality and to reduce the dimensionality of the covariance matrix, and then, finally, the Riemannian tangent space features are extracted. Moreover, it is worth noting that there are two review articles (Congedo et al., 2017; Yger et al., 2017) on the application of Riemannian geometry for BCI systems, which may be an excellent source of information for interested readers.

### Performance Evaluation of BCI Systems

Overall, BCI performance depends entirely on classifier performance (Nicolas-Alonso et al., 2015). For the classification algorithm, the most basic performance measure is classification accuracy. Sometimes, the Kappa metric or the confusion matrix are also used to provide further information on the performance of a classifier (Fatourechi et al., 2008). The sensitivity-specificity pair or precision can be calculated from the confusion matrix. When the classification relies on a continuous parameter, the Receiver Operating Characteristic (ROC) curve, as well as the Area Under the Curve (AUC), is often utilized. Classifier performance is generally computed offline on pre-recorded data utilizing a hold-out strategy: some datasets are set aside to be utilized for the evaluation and are not part of the training dataset. However, some authors also reported cross-validation measures estimated on training data, which may over-rate the performance (Lotte et al., 2018; Raschka, 2018). A number of researchers (Farwell and Donchin, 1988; Iturrate et al., 2009a; Yeom et al., 2014; Obeidat et al., 2015; Ansari and Singla, 2016; Chang et al., 2016; Cao et al., 2017) also reported either the information transfer rate (ITR) or the practical bit rate (PBR) (Farwell and Donchin, 1988). Many articles (Allison et al., 2012; Long et al., 2012a; Li Y. et al., 2013; Cao et al., 2014; Wang H. et al., 2014) used task-specific metrics, including task completion time and the number of successful trials. These metrics are tailored for each BCI paradigm and/or application, and therefore do not allow comparisons between studies.

## Popular EEG-Based BCI Applications

In BCI technology, human brain signals can be detected and translated into device commands for controlling assistive devices. Besides medical applications, the area of this technology has been expanded to non-medical applications. Recently, the possibility of a variety of BCI applications is being investigated by researchers around the world. The most important achievements in EEG-based BCIs include spelling systems, wheelchair control, robot control, mental workload, virtual reality, and gaming, environment control, driver fatigue monitoring, biometrics system, and emotion recognition.

### BCI Wheelchair Control

One of the essential objectives of a BCI wheelchair is to upgrade the life quality as well as the autonomy of people affected by motor neuron diseases (MND), for instance, amyotrophic lateral sclerosis (ALS). This innovation assists the disabled users to operate the wheelchair using their brain activity, granting autonomy to travel through an experimental environment. In 2013, Bi et al. (2013) conducted a survey of BCI-controlled mobile robots, which is partially connected to this field. However, two articles (Fernández-Rodríguez et al., 2016; Al-qaysi et al., 2018; Tariq et al., 2018) have been published that contain extensive reviews on BCI wheelchairs. Four types of EEG control signal are used to handle BCI wheelchairs, which are MI (Li J. et al., 2013; Varona-Moya et al., 2015; Tang et al., 2018), P300 (Rebsamen et al., 2007; Iturrate et al., 2009a; Alqasemi and Dubey, 2010; Shin et al., 2010; Lopes et al., 2013), SSVEP (Mandel et al., 2009; Xu et al., 2012; Mara et al., 2013; Duan et al., 2014; Ng et al., 2014), and hybrid (Li Y. et al., 2013; Cao et al., 2014) signals. The feature extraction methods are quite heterogeneous; however, CSP is the most used EEG feature in BCI wheelchair applications (Li J. et al., 2013; Li Z. et al., 2013; Cao et al., 2014; Wang H. et al., 2014; Zhang R. et al., 2016). Other researchers used methods such as PSD (Varona-Moya et al., 2015; Tang et al., 2018), FFT (Duan et al., 2014), logarithmic band power (Arabnia and Tran, 2011; Duan et al., 2014), signal averaging techniques (Alqasemi and Dubey, 2010; Shin et al., 2010; Zhang R. et al., 2016), the amplitude of the target frequency (Mandel et al., 2009; Mara et al., 2013; Ng et al., 2014), CCA (Xu et al., 2012; Duan et al., 2014), and other methods (Rebsamen et al., 2007; Iturrate et al., 2009a; Lopes et al., 2013). With regard to the classification techniques, the most widely used algorithm is SVM (Rebsamen et al., 2007; Shin et al., 2010; Li J. et al., 2013; Li Y. et al., 2013; Zhang R. et al., 2016), followed by LDA (Iturrate et al., 2009a; Cao et al., 2014). Performance evaluation is the most challenging part in BCI research, as it is the most heterogeneous area. However, the most common metrics reported are success rate, classification accuracy, information transfer rate, path length, time required, path length optimality ratio, time optimality ratio, number of user commands, and number of collisions. Cao et al. (2014) used the highest number of metrics, namely ITR, CA, Collision, Time Required, Useful Command, Useless Command, and Stopping Task Time to evaluate their research. Table 3 shows a summary of some articles regarding EEG-based wheelchair BCI.

**Table 3:** Summary of EEG-based BCI wheelchair studies.

**References**	**No. of subjects**	**EEG control signal**	**No. and types of control command**	**EEG features**	**Classification algorithm**	**Performance evaluation**
Cao et al. (2014)	3	MI+SSVEP	8; Left, Right, Forward, Acceleration, Deceleration, Uniform Velocity, Turn ON, Turn OFF	CSP for MI; CCA for SSVEP	RBF SVM	ITR: 295.20; CA: 90.63%; Collision: 0; Time required: 370 ± 41; Useful command: 5 ± 3; Useless command: 2 ± 2; Stopping task time: 35 ± 4;
Iturrate et al. (2009a)	5	P300	18; Fifteen Locations, Left, Right, Validate selection	Moving average technique	Stepwise LDA (SWLDA)	Success rate: 100%, Time: 659 s, Path length: 39.3 m.
Long et al. (2012a)	2	MI+P300	4; Left, Right, Acceleration, Deceleration	CSP	LDA	Accuracy: 100%, Path length (pixel): 2843.46 ± 105.41, Time: 84.42 ± 4.63 s, Collisions: 0.
Mara et al. (2013)	9	SSVEP	4; Right, left, Forward, Stop	PSD	Decision tree	Success rate: 83 ± 15%, ITR: 70.3 ± 28.8 bits/min.
Li J. et al. (2013)	3	MI	3; Right, Left, Forward	CSP	SVM	Success rate: 82.56%
Li Y. et al. (2013)	5	P300+SSVEP	4; Forward, Stop, Turn ON, Turn OFF	Statistic average Minimum energy combination	SVM	Task duration: 4.30 s/command; TPR: 14.18 event/min, FPR: 0.49 event/min, ITR: 21.11 bit/min.

### BCI Cursor Control

The first attempt to control a cursor by EEG signal was described in Wolpaw et al. (1991). Here, vertical movement of a cursor on a video screen was maintained by changing mu-rhythm amplitude such that the cursor was moved upward by large amplitude mu-rhythm, while downward movement required small mu-rhythm amplitudes. The second experiment conducted by some of the same authors (McFarland et al., 1993) achieved a target hit rate of 54.85%. The promising result obtained encouraged other researchers to develop this research further. Single control signals and hybrid control signals have both been suggested. Li et al. (2010) presented a BCI that enabled the subjects to control vertical movement as well as horizontal movement through P300 and ERD activity, respectively. The subjects could hit one of the four targets with hit rates between 82 and 96%, with average selection times between 25 and 26 s. In their second (Long et al., 2012b) experiment, the trial duration and average accuracy of the target selection were 18.19 s and 93.99%, respectively. The overall outcomes of the experiments were excellent, and the subjects could move the cursor diagonally by executing both sorts of activities simultaneously. However, the cursor control achieved by this system is not continuous. Allison et al. (2012), meanwhile, introduced MI/SSVEP-based hybrid BCI for simultaneous cursor control in two dimensions. The features and classification techniques utilized in this application are heterogeneous. Table 4 shows a summary of some recent investigations in the domain of BCI cursor control.

**Table 4:** Summary of EEG-based BCI cursor control studies.

**References**	**No. of subjects**	**Control signal**	**No. and types of control command**	**EEG features**	**Classification algorithm (CA)**	**Performance evaluation**
Serdar Bascil et al. (2015)	2	MI	2; Left/Right	BP	PNN	CA: PNN: 93.05%
Long et al. (2012b)	11	MI+P300	4; Left, Right, Up, Down	CSP	SVM	Success rate: 93.99% Duration per trial: 18.19 s
Bascil et al. (2016)	5	MI	4; Left, Right, Up, Down	PSD	SVM	CA: 81.22%;
Chakladar and Chakraborty (2018b)	1	MI	4; Left, Right, Up, Down	PSD	DB-Scan	Execution time: 4.663 min, Success rate: 70.36%

### BCI Spellers

An excellent review article (Rezeika et al., 2018) have been published where almost all types of recent BCI spellers have been summarized. Table 5 tabulates the methodology that has been applied along with the performance of some recent EEG-based BCI speller system. In 1988, Farwell and Donchin (1988) presented a P300 speller is known as the matrix speller. It was the first BCI speller and had a maximum accuracy of 95% and a speed of 12 bits/min. An adjacency problem arises in the matrix speller, in that it is difficult to identify a target with a lot of similar objects surrounding it. This problem was avoided by a random-set representation and edges paradigm (Obeidat et al., 2015). Another sort of P300 base speller flashed up a familiar face over a character to improve the speed of spelling (Kaufmann and Kübler, 2014). The checkerboard paradigm (CBP) was presented in Townsend et al. (2010) for avoiding the adjacency-distraction problem and double flash issues. This paradigm achieved better accuracy and was more comfortable than a row-column paradigm (RCP) in ALS patients. A different sort of P300 base paradigm, namely a gaze-independent block speller, was also introduced (Pires et al., 2011), which has the ability to be utilized without ocular movement. Additionally, this paradigm creates almost the same information transfer rates as standard to a row-column speller. Acqualagnav et al. (2010) proposed rapid serial visual presentation (RSVP) with the aim of forming an efficient gaze-independent ERP speller. The subject showed better performance with the colored letters than with monochrome ones. The accuracy of the RSUP speller is better than that of the matrix speller. It is worth noting that one of the earliest high-speed SSVEP-based BCI spellers is the Bremen BCI speller (Volosyak et al., 2009). The efficacy of this speller was examined on both healthy and disabled people. The average ITR reported was 25.67 bits/min, with an accuracy of 93.27%. Furthermore, Cao et al. (2011) proposed a multi-phage SSVEP-based speller system that allows the input of 42 characters comprising letters, digits, and symbols. The mean ITR and mean accuracy of this speller are 61.64 bits/min and 98.78%, respectively. However, this speller did not include MND patients as subjects to test the system. In the same vein, Nakanishi et al. (2018) proposed a multi-target one-phase SSVEP speller by achieving an 89.83% accuracy and 325.33 bits/min ITR. In general, it is worth noting that a higher number of targets in SSVEP-based BCI is shown to increase the spelling speed but also to increase eye fatigue and target misclassification.

**Table 5:** Summary of EEG-based BCI speller studies.

**References**	**No. of subjects**	**Control signal**	**Method**	**Classification algorithm**	**Typing speed**	**Success RATE**
Obeidat et al. (2015)	14	P300	Stimuli variation	Bayesian BLDA	13.7 bits/min	93.3%
Cao et al. (2017)	3	MI	Oct-O-spell	SVM	67.33 bits/min	98.23%
Ansari and Singla (2016)	20	SSVEP	Multi-phase spellers	SVM	13 chars/min	96.04%
Chang et al. (2016)	10	SSVEP+P300	Hybrid speller	CCA, SWLDA	31.8 bits/min	93%
Käthner et al. (2015)	19	P300	Familiar faces and symbols	SWLDA	15.85 bits/min	95%

A Hex-O-Spell, which depends on imaginary movement, has also been reported on Blankertz et al. (2006). The speller was demonstrated to offer higher performance than the conventional matrix speller. A recent MI-based speller, namely Oct-O-Spell, was introduced in Cao et al. (2017), involving an octagon divided equally into eight sections. These sections contained a total of 26 letters, characters, digits, or symbols. The interface showed a similar performance to hybrid BCI spellers. Similarly, a hybrid BCI speller based on SSVEP and P300 was presented in Chang et al. (2016), again featuring an octagon divided equally into eight sections. Each section consisted of a total of 26 letters, characters, digits, or symbols. The interface showed a similar performance to hybrid BCI spellers. The authors of Nguyen et al. (2018) proposed a high-speed BCI spelling system using eye closure and double-blink EEG by means of an SVM classification algorithm. It was demonstrated from the investigation that the proposed system is able to achieve an average classification accuracy of 92.5% with an ITR of five letters per minute.

### BCI Biometrics

Biometrics is the process of identifying one individual among others by biological means. Biometrics, including iris, face, and fingerprint recognition, is frequently applied to avoid security breaches. Nonetheless, the possibility of imitating, replicating, or stealing original information has made these tools unreliable. As a result, there has been a growing interest in finding a better biometric system and brain activity-based biometrics. An EEG system-based biometric has been identified to have the advantage of being quite impossible to mimic (Alariki et al., 2018). Using 15 human participants, Ruiz Blondet et al. (2015) studied the stability of EEG brainwaves over a 6-month period. Based on their findings, it was shown that the accuracy of EEG signals for biometric systems and the stability of human brain activities could remain stable over a long time. Bashar et al. (2016) proposed a method for human identification using EEG signals. The authors used a bandpass FIR filter to remove noise and then divided the EEG signals into two sections. Multi-scale Wavelet Packet Statistics (WPS), Multi-scale Shape Description (MSD), and multi-scale Wavelet Packet Energy Statistics (WPES) were utilized as the features in this method, which were in the time-frequency domain, and SVM was the classifier. A true positive rate of 94.44% in the aforesaid method was achieved using nine subjects in an experiment. Ruiz-Blondet et al. (2016) proposed an ERP-based highly accurate biometric recognition system designed to extract unique individual responses from the brain. The authors reportedly achieved 100% identification accuracy using 50 subjects. Table 6 shows a summary of some BC-based biometrics and other related research.

**Table 6:** Summary of EEG-based BCI biometrics research.

**References**	**Application**	**No. of subject**	**EEG features**	**Classification algorithm**	**Performance evaluation**
Pham et al. (2013)	Person authentication system	9	PSD	SVM	Multiple matched policy is highly secured
Bajwa and Dantu (2016)	Cancelable biometrics-based key generation	Data-1: 7, Data-2: 120	DFT, DWT	SVM	CA: Data-set-1: 98.46%; Data-set-2: 91.05%
Hu (2018)	EEG-based gender recognition	28	Fuzzy entropy	Vote classifier	Average accuracy: 99.8%
Nguyen et al. (2017)	Cryptographic key generation	125	PSD	Enroll and KeyGen	Success rate: 99%
Bashar et al. (2016)	Human identification	9	MSD, WPS, WPES	SVM	The highest accuracy: 94.44%
Ruiz-Blondet et al. (2016)	Biometric identification	50	Average ERP	SVM	Accuracy: 100%

### BCI Emotion Recognition

Data acquisition from brain signals connected to human emotion is one of the core steps toward emotional intelligence. Picard stated that emotions play a vital role in rational decision-making, learning, and perception, as well as in a variety of functions (Picard, 2003). The identification of emotion changes from EEG signals has recently achieved attention among BCI researchers in the process of developing different BCI devices. There are three excellent review articles (Al-Nafjan et al., 2017; Soroush et al., 2018; Xu T. et al., 2018) on EEG-based emotion recognition. These articles might be of help to the interested readers to further have an insight of the EEG-based emotion recognition.

A number of emotion-recognition studies have been carried out by BCI researchers in the last 20 years. Table 7 summarizes some recent emotion recognition approaches in terms of the number of subjects, stimulation, emotion types, feature extraction, classification method, and performance. Mu Li and Bao-Liang Lu (2009) utilized EEG signals for emotion recognition in response to emotional pictures. Their study gained a recognition rate of 93.5% for two emotional states. Petrantonakis and Hadjileontiadis (2010) presented a user-independent emotion recognition strategy that achieved an 83.33% recognition rate for six emotion categories. Wei et al. (2017) proposed a combination of features for achieving higher accuracy. The experimental outcomes showed that the combination of Power Spectral Density (PSD), Signal Power (SP), and Common Spatial Pattern (CSP) as the features achieved the highest accuracy of 86.83% with LDA as the classification algorithm, whereas, the accuracy of individual features was 64.73%. Chakladar and Chakraborty (2018a) introduced a correlation-based subset selection technique for dimensional reduction and used higher-order statistical features (mean, skewness, kurtosis, etc.) for classification. The authors classified four classes of emotion through employing an LDA algorithm, and an overall accuracy of 82% was attained. Moreover, Anh et al. (2012) proposed an emotion identification scheme to identify 2 valence classes and 2 arousal classes, which resulted in a combination of 4 fundamental emotions (happy, sorrowful, angry, and relaxed) and the neutral state. The authors affirmed the fractal dimension for feature selection and SVM as a classifier, where the average accuracy across all subjects was 70.5%. Liu Y. et al. (2017) designed a movie-induced feelings recognizer using EEG. This framework reached 92.26% accuracy in recognizing neutrality from high arousal with valence emotions and 86.63% to classify negative from positive emotions. The authors similarly classified 3 positive emotions and 4 negative emotions with 86.43 and 65.09% accuracy, respectively. Here, STFT was for feature extraction and SVM for classification. Another study reported the recognition of three classes of human emotion, namely sorrowful, excited, and relaxed, in real time using Wavelet and Learning Vector Quantization (LVQ) with an accuracy of 72–87% (Djamal and Lodaya, 2017). Moreover, a group of features, namely power, standard deviation, variance, and entropy, were classified by utilizing the k-NN algorithm. Happiness, anger, and calm were categorized in Kaur et al. (2018). Here, the fractal dimension feature was classified by utilizing RBF SVM with 60% accuracy. In another study, a human-vehicle collaborative driving (HVCD) simulation system was designed by integrating visual information and human intentions to achieve a comfortable and safe driving experience (Li et al., 2018). The average accuracy and ITR achieved were 91.1% and 85.80 bit/min, respectively.

**Table 7:** Summary of EEG-based BCI emotion recognition studies.

**References**	**No. of subjects**	**Stimulation**	**Emotion types**	**EEG features**	**Classification algorithm**	**Performance evaluation**
Wei et al. (2017)	12	Pictures	Positive and negative	PSD, SP, CSP	LDA	Average accuracy: 86.83%
Wang X.-W. et al. (2014)	6	Movie clips	Positive and negative	PSD	LDA	CA: 91.77%
Liu Y. et al. (2017)	30	Movie clips	Joy, anger, fear, sadness, disgust, and neutrality	PSD	LIBSVM	Average accuracy: 89.45%
Kaur et al. (2018)	10	Video clips	Calm, angry, and happy	FD	RBF SVM	CA: 60%
Özerdem and Polat (2017)	32	Music clips	Positive and negative	DWT	MLPNN	CA: 77.14%
Pan et al. (2016)	6	Photos	Happiness and sadness	CSP	SVM	CA: 74.17%
Djamal and Lodaya (2017)	10	Music	Excited, relaxed, and sad	WT	LVQ	CA: 87%
Murugappan (2011)	20	Video clips	Disgust, happiness, fear, surprise, and neutral	Entropy	K-NN	CA: 82.87%

### BCI Virtual Reality and Gaming

This section has been inspired by some excellent review articles on BCI-based VR and games that are reported in Cattan et al. (2018), Ahn et al. (2014), and Kaplan et al. (2013). The research on BCI systems for healthy subjects has attracted considerable interest. The prototypes of BCI-based video games in existence are based on three BCI paradigms: steady-state evoked potential (SSVEP), P300 event-related potential (ERP), and mental imagery (MI). Table 8 shows a summary of some recent EEG-based virtual reality and gaming systems. Finke et al. presented a P300-based BCI game known as Mind Game in which the user moves a character from one field to another on a game board (Finke et al., 2009). For Mind Game, the authors reported a 66% mean accuracy (specifically, this was the rate at which the correct target was selected out of 12 possible targets). Other P300-based BCI games have also been proposed (Mühl et al., 2010; Congedo et al., 2011; Angeloni et al., 2012; Ganin et al., 2013).

**Table 8:** Summary of EEG-based BCI gaming and VR studies.

**References**	**No. of subjects**	**Control signal**	**EEG feature**	**Classification algorithm**	**Performance evaluation**
Kreilinger et al. (2016)	10	MI	BP	LDA	“Upper 10%” MI detection rates: >70%
Bonnet et al. (2013)	10	MI	CSP	LDA	CA: >70%
Maby et al. (2012)	2	P300	Shannon entropy	LDA	Average accuracy: 82%
Djamal et al. (2017)	10	MI	FFT	LVQ	Average accuracy: 70%

Some famous video games, including Pong, Pacman, and similar games, can be played with motor imagery (Krepki et al., 2007). The Pacman produces one step every 1.5–2 s with the aim of giving the gamer enough time to perform a control command. In another study, a pinball game was developed in order to illustrate that it is possible to successfully apply non-invasive recording techniques for complex control tasks (Krauledat et al., 2009).

Moreover, external evoked potentials have been utilized for game implementations. Middendorf et al. (2000) designed a simple flight simulator that is controlled by a BCI based on Steady-State Visual-Evoked Response (SSVER). This simulator was very modest, and only two control actions were possible. The position could only be moved to the left or right. Two methods were tested over the airplane control trials. On the other hand, the control command (right or left) was detected according to the strength of the SSVERs. The selection was identified by reference to the frequency of SSVER. The results of the trials with able-bodied participants showed that the last one was preferred because it required little or no training since the system capitalized on naturally occurring responses. Lalor et al. (2005) proposed a game named Mind Balance in which healthy subjects needed to hold a tightrope walker in balance. The application was based on SSVEP, which is generated as a response to phase-reversing checkerboard patterns.

A final interesting example of this application was recently presented at the Cybathlon 2016, a competition for participants with disabilities who compete against each other using assistive technologies. In the BCI discipline, 11 participants with tetraplegia competed against each other in a virtual environment where their avatars raced along a virtual obstacle course (Novak et al., 2018). Since external visual stimuli were not allowed at the Cybathlon, the participants could not make use of SSVEPs and P300; instead, they relied on motor control and/or mental imagery to control their avatars. As expected, the results varied strongly between the 11 participants, with the best participant completing the race in the 90 s and the worst completing it in 196 s. In this competition, every team used gelled electrodes, indicating that they did not consider dry or water-based electrodes reliable enough for use in uncontrolled environments. Similarly, every team used laboratory-grade EEG amplifiers, suggesting that no team trusted consumer-grade devices to provide sufficiently good performance. Furthermore, the competition emphasized the importance of effective BCI training for the users, as the teams all had very different participant-training strategies and the winning team stated that their effective BCI training regimen likely had a major effect on their success.

### BCI Robotic Arm

Because of the advances in robot control (RC) systems, they are playing an increasingly important role in a wide range of fields. The relationship between humans and robots has become increasingly intimate, and many human-robot collaboration systems have been developed. However, it is hard for a disabled person to operate a robot because of their loss of motion capacity or reduced sensing ability (Yang C. et al., 2018). Many studies have been dedicated to solving this problem (see Table 9). Yang C. et al. (2018) presented a shared control system by combining an SSVEP-based BCI and visual servoing (VS) technology to enable mind control of a robot manipulator. To enhance the intelligence and accuracy of the shared control system, the authors proposed an adaptive color adjustment for object detection, the least squares method (LSM) for camera calibration, and the coordination of task motion and self-motion (CTS) for obstacle avoidance. However, the authors tested the system using only two healthy subjects. Furthermore, Bousseta et al. (2018) proposed a preliminary outcome for the movement control of a robot arm for four directions: left, right, up, and down through mental tasks. Spectral analysis with FFT transform was combined with a PCA strategy to produce optimal features to feed into an RBF Kernel SVM classifier aimed at distinguishing the four movements. The experiments performed by four volunteers produced an average accuracy of 85.45%. A hybrid BCI system consisting of SSVEP and MI was proposed in Duan et al. (2015) to provide control commands to a brain-actuated robot. The SSVEP responses were utilized to tell the robot to move forward, turn left, and turn right, whereas the MI was utilized to perform the grasp motion. The authors also developed a visual servo module to perform the grasping with higher accuracy.

**Table 9:** Summary of EEG-based BCI robotic arm studies.

**References**	**No. of subjects**	**Control signal**	**EEG feature**	**Classification algorithm**	**Performance evaluation**
Yang C. et al. (2018)	2	SSVEP	FFT	CCA	Five tasks performed
Hortal et al. (2015)	2	MI	FFT	SVM	CA: 70%
Bhattacharyya et al. (2015)	11	MI	MFDFA	ANFIS	Success rate: >60%
Bousseta et al. (2018)	4	MI	FFT	RBF SVM	Success rate: 85.45%
Roy et al. (2016)	5	MI	WT, PSD	SVM	CA: 75.77%

### BCI Environmental Control

An important application of EEG-based BCIs is environmental control, which can improve the quality of life and increase the independence of paralyzed patients. Various EEG-based environmental control systems have been developed in recent years.

Aloise et al. developed a P300-based BCI home electronics control system that included a DVD player, electric lights, etc. (Aydin et al., 2018) where the subjects suffered from chronic neurological disorders. Shyu et al. (2010) proposed a steady-state visual evoked potential (SSVEP)-based BCI multimedia control system. Specifically, four flickering buttons were utilized to perform four different control commands: play or pause the selected multimedia file, scroll through displayed items, stop the multimedia system, and adjust the volume up and down. In another study, the authors proposed the application of an SSVEP-based BCI hospital bed nursing system (Kleber and Birbaumer, 2005) that enabled the users to move the entire bed up and down and control the power of the massaging cushion. In a study carried out by Corralejo et al. (2014), several patients with different degrees of motor impairment were able to operate home electronics, including a TV, DVD player, and lights, via a P300-based BCI. Edlinger and Guger (2012) suggested a non-invasive subject dependent P300 stimulus-based BCI system that provides the subject with a stimulus and records his reaction as an input. The user is provided with a GUI with various icons representing different tasks like turning a light on/off, opening a door/window, switching TV channels, etc. In the worst-case scenario, this system obtained 30% accuracy with 12 participants, whereas, in the best-case scenario, a 100% accuracy was achieved from one of the subjects.

In another perspective, Carabalona et al. (2010) suggested a non-invasive visual P300-based BCI system for physically impaired users to control a smart home environment. The user is provided with a 6 × 6 matrix of icons. Each icon represents a command related to an everyday device. The icons flash on the computer screen one by one. Once the desired icon is reached, a peak is observed in the neural signals of the user. This peak is considered to be icon selection. The system was tested using four participants, and the accuracy rate observed varied from 33 to 100% among different users. Kim et al. (2013) also suggested a non-invasive P300 stimulus-based BCI system to switch TV channels from a viewing distance of 3 meters and a 46-inch TV screen. A total of eight subjects were provided with a visual stimulus in the form of a flashing green cursor in the top left corner of each channel icon. Once the desired channel was reached, a peak in the subject's neural signals was considered as input for channel selection. An average of 92.3% accuracy was attained from the system. In two other studies (Edlinger and Guger, 2012; Lin et al., 2014), the authors proposed non-invasive subject-dependent BCI to control electrical home appliances. Two physiological states (drowsiness or alertness) of users were used and translated into commands to interact with different electronic appliances. Similarly, Akman Aydin et al. (2015) suggested a region-based selection paradigm for a smart home control system for physically impaired users. The proposed system is a non-invasive P300 stimulus-based BCI system that flashes each region five times on the screen to invoke a response from the user. Upon acquiring a peak in the neural signals of the subject, the system considers it as a selection command for that particular region. The proposed system was able to achieve 95% accuracy for 49 household tasks using five subjects without any physical impairment. Masood et al. (2016) suggested a non-invasive BCI that uses the blink of an eye as a control input to interact with home appliances. The selection and control of the device are performed using a GUI. The system has the potential to be enhanced by adding more devices. However, the currently proposed system has only 70% accuracy. Table 10 summarizes some recent findings with regard to EEG-based BCI environmental control.

**Table 10:** Summary of EEG-based BCI environmental control studies.

**References**	**BCI application**	**Control signal**	**No. of subject**	**Method**	**Performance evaluation**
Shyu et al. (2013)	Hospital bed nursing system	SSVEP	15	FPGA	Accuracy: 92.5%, ITR: 5.22 s/command
Zhang et al. (2017)	Environmental control system	ERP	3	Classifier: BLDA	Accuracy: 89.2%
Aydin et al. (2018)	Environmental control system	P300	10	Classifier: LDA	Accuracy: 93.71%
Kosmyna et al. (2016)	Control of a smart home with a BCI	P300, SSVEP	12	Minimum Distance Classifiers	Average accuracy: 81–77%

### Recent Achievements and Innovations in EEG-Based BCIs

**Figure 4:**
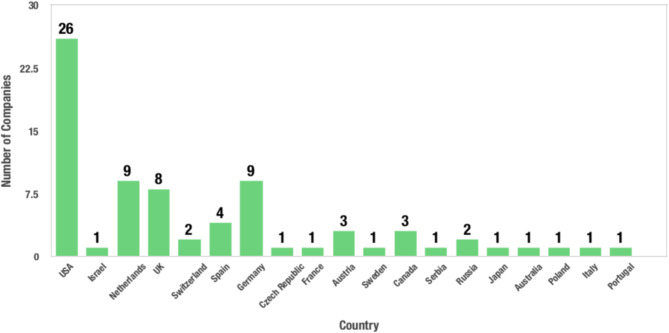
Distribution of BCI companies around the world.

In its early days, BCI technology was regarded as unattractive for genuine scientific investigation, primarily owing to its restricted resolution and the unreliability of the information from the brain as well as its high variability. Furthermore, the data acquisition of such technology was somewhat expensive. Therefore, brain activity research was typically constrained to medical use or exploration of brain functions in the laboratory. However, this scenario has changed dramatically over the past two decades due to advances in technology, which have brought down its associated costs to some degree. Meanwhile, BCI research has expanded to non-medical applications as well. Over the last 15 years, the number of studies regarding BCI has increased substantially. Figure 4 depicts the number of companies that manufacture BCI-based products around the globe^2^. The two most well-known technology providers for BCI devices are Neurosky and Emotive.

The leading social media platform, Facebook, funded a research project at UCSF that aims to restore the communication ability of disabled people through their thoughts at a speed of 100 words per minute. Eventually, this company is planning to launch an EEG headset that lets users control music or interact in virtual reality using their thoughts. Some findings of this project were published in Moses et al. (2019), where the authors demonstrated that high-resolution recordings directly from the cortical surface can be used to decode both perceived and produced speech in real time. By integrating what participants hear and say, they leveraged an interactive question-and-answer behavioral paradigm that can be used in a real-world assistive communication setting. Together, these results represent an important step in the development of a clinically viable speech neuroprosthesis.

SpaceX and Tesla CEO Elon Musk founded Neuralink, which is aiming to design ultrafine threads (more tenuous than a human hair) that can be embedded into the brain to recognize neural activity. It builds arrays of small and flexible electrode “threads,” with as many as 3,072 electrodes per array distributed across 96 threads Musk (2019). This company has also built a neurosurgical robot that is capable of inserting six threads (192 electrodes) per minute. Each thread can be individually inserted into the brain with micron precision for the avoidance of surface vasculature and to target specific brain regions. The electrode array is packaged into a small implantable device that contains custom chips for low-power onboard amplification and digitization: the package for 3,072 channels occupies <23 × 18.5 × 2 mm^3^. The size and composition of the thin-film probes are a better match for the material properties of brain tissue than commonly used silicon probes and may, therefore, exhibit enhanced biocompatibility (Chung et al., 2019). A single USB-C cable provides full-bandwidth data streaming from the device, recording from all channels simultaneously. This system has achieved a spiking yield of up to 70% in chronically implanted electrodes.

Jiang et al. (2019) have presented BrainNet, which is the first multi-person non-invasive direct brain-to-brain interface for collaborative problem-solving. The interface combines EEG to record brain signals and transcranial magnetic stimulation (TMS) to deliver information non-invasively to the brain. Among the three human subjects, two subjects are designated as “Senders” whose brain signals are decoded using real-time EEG data analysis. The decoding process extracts each Sender's decision about whether to rotate a block in a Tetris-like game before it is dropped to fill a line. The Senders' decisions are transmitted via the Internet to the brain of a third subject, the “Receiver,” who cannot see the game screen. The Receiver integrates the information received from the two Senders and uses an EEG interface to make a decision about either turning the block or keeping it in the same orientation. Five groups, each with three human subjects, successfully used BrainNet to perform the collaborative task, with an average accuracy of 81.25%. This result points the way to future brain-to-brain interfaces that enable cooperative problem solving by humans using a “social network” of connected brains.

Boston area company Neurable has created a brain-controlled game called Awakening, the central character of which is a child with telekinetic powers. The character is set the task of escaping from a laboratory by using mind power to pick up toys such as a balloon dog and rainbow rings. Players wear a headband studded with electrodes that connect to a virtual reality headset. Their brain signals are picked up and analyzed by software that determines how the character will move. Players are then able to train their brains to produce the right signals to pick up the toys (iHuman, 2019).

Over the last two decades, evidence has accumulated on the capacity of neural interfaces such as those using transcranial direct current stimulation (tDCS) to enhance performance in cognitive areas such as working memory and attention as well as in physical activities such as cycling (Ke et al., 2019). tDCS involves the use of a headset and electrodes that are typically contained in sponge bags, with saline solution used to conduct electricity from the electrodes to the scalp (Cincotti et al., 2003). Adverse effects are rare—users have reported mild tingling sensations and occasionally headaches or fatigue. One study showed how tDCS improved the ability of US Air Force personnel to “multitask” (Nelson et al., 2016). The participants, all stationed at the Wright-Patterson Air Force Base in Ohio, USA, were asked to monitor and respond to four independent tasks on one computer screen. Their tasks were to: keep dial markers centered in a “system monitoring” box; change the communications channel frequencies as requested by an audible prompt; keep a target centered in a “targeting” box, and keep fluids moving in a “resource management” box by turning tanks on and off. The 10 participants who received active tDCS stimulation from the headsets, provided by Wales-based company Magstim, performed about 30% better than those who did not.

## Current Challenges and Directions

In the past few years, substantial BCI research has been performed to invent some potential assistive technologies. From the current status of BCIs, it can be predicted unquestionably that BCI technology will very soon be launched in the market. In fact, a few commercial BCI appliances have already been launched in the market. A remarkable program, namely the BNCI Horizon 2020 project, has proposed a future agenda regarding BCIs. However, some crucial issues and challenges exist in every component of the BCI paradigm, and these issues should be addressed by the BCI community to make further advances in BCIs.

### Issues in EEG Modalities for BCI Applications

The most preferred EEG modalities, namely MI, SSVEP, and P300, are continuously facing signal processing issues, especially the identification of the most applicable approaches for feature extraction and feature reduction. This is primarily owing to the nature of the EEG signals, namely extremely non-linear, non-stationary, and artifact-prone. Other notable issues involve data fusion, in particular how the data from numerous electrodes are merged to be able to lessen the data dimensionality as well as to make improvements to the classification results. It is evident that once individuals have been taught properly, MI strategies often deliver remarkable outcomes. For an MI-based BCI to be handled by a targeted user, plenty of training trials are needed from the targeted user, causing the calibration period to be unacceptably long for a realistic model. Thus, investigations should be concentrated on cutting down the calibration period as well as effective training strategies. P300 shows greater average ITRs, and it does not call for a training process, although the degree of severity and variety of the disease may considerably affect the performance of this modality. Nevertheless, a good number of studies have also found that even individuals with LIS are capable of handling a P300-based BCI for long durations. However, with regards to ITR, it is worth noting that the healthy individual group yields higher bit rates than disabled subjects in practically all the previous findings regarding P300-BCI (Lazarou et al., 2018). The method of stimulation is so difficult that the experimental process could not be carried out by the patients. Moreover, a wide range of instructions in a P300-based BCI system increases the number of trials, which, in turn, causes reduced overall performance. The coupling of the generic models with online training could be an excellent alternative to reduce the calibration period and enhance P300-based BCI system performance together with consumer satisfaction (Jin et al., 2020). The SSVEP approach obviates the calibration or subject training. Hence, this type of speller should be faster in comparison with P300 spellers. However, a certain number of individuals generate extremely poor SSVEP responses, which is tough to explore. Therefore, a hybrid approach could be an excellent alternative to the use of a single EEG modality. Further investigations are necessary in order to identify which EEG modality is the most appropriate type for BCI application. For instance, MI-based approaches are considered the most appropriate to handle a prosthetic arm or leg. However, in the operation of digital radio, an SSVEP-based approach with a 4-option menu including volume-up, volume down, station-up, and station-down options may be much intuitive than an MI-based technique with imagined movements being associated with television controls. Intuitiveness should be a crucial factor at the time of the initial decision stage when selections of technology and BCI category are being made. Additional investigations are also needed to figure out the more appropriate feature set and classification frameworks for specific EEG modalities. Research with regards to features and classifiers also need emphasize figuring out the optimal options to be employed for individuals affected by CNS injury.

### Issues With EEG Headsets

The quality of EEG data for BCI application mostly depends on the EEG headset. There are some issues regarding EEG headsets that need to be resolved. First of all, most of the EEG headsets require gel or liquid on electrodes, which are very uncomfortable to the user. For practical applications, users prefer dry electrodes, as it is not mandatory to use any conductive gel between the scalp skin and electrode pad. However, it is a matter of open debate as to whether this type of electrode offers identical signal properties. It has been reported that the EEG from dry electrodes contains considerably more artifacts and noise than wet electrodes, whereas another study reported that the signal properties are almost identical for wet and dry electrodes. Hence, an in-depth investigation should be carried out to further validate the efficacy of dry electrodes. It is worth noting that newly proposed water-based EEG electrodes are now being investigated. In Mihajlovi and Peuscher (2012), the authors pointed out that the performance of dry and water-based electrodes with shorter hair is comparably superior to gel-based electrodes. They also recommended continuing further investigation for the refinement of electrodes to make them applicable with longer hair and further suggested that dry and water-based electrodes have the potential to replace gel electrodes. To date, headsets with dry electrodes are available in the market, but these contain very few electrodes, and the procedure has not yet been standardized. There are thousands of EEG headsets that have already been launched by different companies for BCI applications. The number of electrodes (for example, 1, 3, 4, 8, 14, 16, 20, 24, 32, or 64) is varied from headset to headset, and these headsets are not compatible with each other. Hence, the minimum number of electrodes for specific EEG modalities should be standardized. High ownership cost of an EEG headset is another challenge in allowing BCI technology to become affordable for the general public around the world (Abiyev et al., 2016). Hence, the reduction of the price of such EEG headsets is much lauded. The portability of the BCI model is also a crucial matter: headsets that transfer data wirelessly permit individuals to move freely, whereas a wired headset somewhat limits movements.

### Lack of Ideal Data Analysis Methods

Although most of the artifact removal algorithms offer good performance, the methods listed in section EEG Data Pre-processing Strategies suffer from different limitations when utilized in a particular EEG-based application. Indeed, some methods are only focused on the detection and removal of particular artifacts. Some methods need reference channels to enhance the accuracy of artifact removal, which is not feasible for some specific applications. ICA-based algorithms can deal with all kinds of artifacts occurring in EEG recordings. Regression and adaptive filters are more feasible choices when the reference channels for specific artifacts are available. Apart from ICA, CCA and its combination with other methods seem to be a good choice for the removal of muscle artifacts. For application to a few channels, EMD, IVA, and its hybrid methods with BSS or WT can be an ideal choice. Moreover, EMD can significantly improve the signal quality by eliminating noise with fewer data (Zhang Z. et al., 2019). However, the requirement for a reference signal limits adaptive filter or regression methods to the removal of particular types of artifact. Wavelet transform fails to completely identify artifacts that overlap with spectral properties. EMD also suffers from the drawback of mode-mixing. Therefore, it is quite difficult to find a single method that is both efficient and accurate enough to satisfy all the conditions perfectly. Thus, one of the future objectives of the effective attenuation of artifacts is to develop an application-specific algorithm with better time-efficiency and accuracy. Also, from the current trend of artifact removal, it can be concluded that future directions will combine machine learning and traditional approaches for effective automatic artifact removal. Apart from that, new artifact removal algorithms for numerous types of artifacts in multiple scenarios still need to be identified.

Regarding feature extraction techniques, a wide range of features have been extracted to figure out the significant information from the EEG. From previous studies, it has been seen that the performance of CSP and its modified algorithms is comparatively encouraging when applied to EEG motor imagery data. In the case of emotion recognition or mental workload classification, spectral features are more suitable, whereas frequency-domain based features perform better with SSVEP data. However, it is too early to state the optimum feature extraction technique for a specific EEG control signal modality.

Based on previous studies, SVM is the most robust classifier for classifying high-dimensionality feature vectors. Sometimes, HMM may deal with a high-dimensionality feature set by analyzing the sequence of feature vectors. In order to avoid computational complexity, a low-dimensional feature vector should be preferred. However, feature reduction or selection algorithms could be considered in the case of high-dimensional feature sets. Several deep learning approaches have been implemented to classify MI, SSVEP, emotion recognition, and ERP data. The architecture of deep networks entirely depends on network structure and input formalization. Previous studies showed that CNN- and RNN-based deep learning approaches outperformed other deep learning methods. Moreover, CNN offered optimum accuracy when time-series values or spectral images were utilized as inputs. Hence, it has been suggested that there should be comprehensive study of the combination of network architectures, especially the structure and number of distinctive layers, for example, RBMs, convolutional layers, fully connected layers, and recurrent layers. Besides network structure, further studies need to be conducted to distinguish how deep learning approaches interpret raw EEG against artifact-prone EEG, as these sorts of studies have not yet been explicitly conducted. In this article, a wide range of classifiers have been surveyed that are evaluated in an offline manner. As every BCI application is essentially an online scenario, the classifiers should be validated online. Moreover, the classifier model should be tested to assure lower computational complexity and calibrated quickly in real-time operation. To design calibration-free BCIs, domain adaptation, and transfer learning can be an effective alternative where the combination of superior feature sets, for example, covariance matrices and domain adaptation algorithms, may enhance the invariance capability of BCIs. The design of a stable estimator of the Riemannian median is an open challenge. This property could make the Riemannian geometry classifiers more robust.

### Performance Evaluation Metrics

It is evident from the previous studies that a variety of performance evaluation metrics are employed to evaluate BCI systems. It is almost impossible to compare the same types of BCI systems when they are evaluated by dissimilar performance metrics. Hence, the BCI research community should recommend a standard and systematic approach or a single metric to quantify a specific BCI application. For example, number of control commands, types of control commands, distance covered, time required, number of collisions, classification accuracy, average success rate, amongst others, should be utilized to evaluate the performance of a BCI wheelchair control. If the same performance metrics are used for a given BCI application, then a direct comparison between different BCI experiments is possible. Not only are training models not liable to limit the overall performance of the system, but testing data can also be utilized. A large number of BCIs are designed to substitute CNS functionality, and these BCIs are actually assessed on healthy individuals inside a controlled laboratory environment. This may misguide to defective outcomes if the targeted users of these systems would be disabled patients; for example, BCI performance seems to be poorer for patients affected by spinal cord injuries when compared with healthy subjects. Thus, researchers should emphasize specifying similar performance evaluation metrics and, in the meantime, ensure system assessment by valid data.

### Trends in Lab-Based BCI Technology

One of the major concerns regarding BCIs is that almost all of BCI experiments have been conducted in a controlled lab, regardless of the realistic environment of the targeted users. Therefore, these EEG data are a crucial factor in the initial evaluation of signal processing approaches as well as the advancement of considerably more robust systems. As pointed out before, heart rate and cortisol may considerably affect the characteristics of brain waves. Outside the lab, different sensory stimulations found in the surroundings, like sounds, movements, and smells, may affect the quality of EEG signals. Hence, during the design of any BCI system, engineers should consider the particular environment where the proposed technology will be employed. For example, the design criteria may not be identical for the operation of home appliances in a home environment as compared to the detection of the attention level of a pilot using EEG while flying a plane. Thus, at the time of system design, it is important to examine the basic criteria of the system, environmental aspects, situation, and target users in-depth.

### Low ITR of BCI Systems

Higher ITR is the primary requirement of any effective BCI system. ITR is the metric that is most employed in the BCI community to assess the performance of BCI prototypes. The ITR of a given BCI system depends upon three criteria: the number of classes, target detection accuracy, and target detection time (Wolpaw et al., 2002; Ramadan and Vasilakos, 2017). The target detection accuracy can be boosted with the aid of the enhancement of the Signal-to-Noise Ratio (SNR). Several strategies are usually employed in the preprocessing phase in order to increase the SNR, as described in section EEG Control Signals Used in BCI Applications. Once high ITR is achieved, more sophisticated applications may be developed by increasing the variety of classes. A variety of stimulus coding strategies, for instance, CDMA, TDMA, and FDMA, have already been adapted for BCI systems (Bin et al., 2011; Jin et al., 2011). In order to code the intended stimuli, TDMA has been utilized with P300, whereas CDMA and FDMA have been employed in those BCIs that deal with VEP. Finally, minimizing the target recognition period, which assists in boosting the ITR, is another crucial aspect of BCIs. To accomplish this, adaptive approaches, namely “dynamic stopping,” may be an excellent alternative. Moreover, single-trail classifications using machine learning frameworks and optimized stimulus demonstrations can also contribute to reducing the target recognition period (Schreuder et al., 2013).

### Commercialization of EEG-Based BCI Technology

The main consumer of BCI technology is this particular group of disabled patients, and commercialization is the only way to spread this technology all over the world. Hence, the BCI research community should identify the actual causes that prevent the commercialization of this technology. To further illustrate the obstacles that are preventing the commercialization of BCI technology, Vansteensel et al. (2017) supplied a set of questions to more than 3,500 BCI researchers in the field of EEG or EMG technologies throughout the world. Almost all of the experts were confident that a particular BCI application would be launched to the market within the next 5–10 years. This survey suggests that upcoming research ought to concentrate on sensor development as well as the overall system performance of non-invasive BCIs. Kristo et al. (2013) also concluded that the BCI research community should focus on boosting ITR (Speier et al., 2013) and exploring the signal processing platform. In addition to these, there are also some major issues that prevent the commercialization of an EEG-based BCI system among the targeted users. First of all, existing BCIs cannot be handled by disabled patients. Expert assistance is mandatory to set up the signal-receiving electrodes of current BCIs. Moreover, almost all of the BCI devices are still under investigation and, hence, are not readily available for home-usage. Therefore, future BCI systems should cater for disabled patients lacking any assistance from experts. BCI illiteracy is an additional barrier to the widespread use of EEG-based BCI systems. When an individual is unable to operate the BCI device due to low-quality brain signals being produced, this phenomenon is regarded as BCI illiteracy. EEG signal quality may be improved with the aid of a collaborative co-learning strategy that gives the end-user audio or visual feedback. Continuous use of BCIs makes frequent use of specific neural pathways (Padfield et al., 2019), and additional study needs to figure out the possible health risks or variations in brain functionality due to such prolonged exposure. The BCI system should be similar to other ideal off-the-shelf technologies with regards to its ease-of-use. The BCI devices should also be user-friendly and have in-built safeguards to prevent untoward situations. Furthermore, advanced BCI technology should also be capable of providing stable results when it is used in multisensory environments such as in a noisy family home. Table 11 lists the essential features that enable the commercialization of BCI systems. These features are deemed necessary to increase the quality of BCI systems (Miralles et al., 2015).

**Table 11:** Essential features for BCI systems.

**Key feature**	**Description**
Effectiveness	The BCI system should be really helpful to users
Robustness	The system must be stable during regular use and robust with respect to anomalies
Quick operation	Task execution time should be as low as possible
More functionality	The system should allow the user to perform as many tasks as possible to increase the autonomy of the user
Safety	The system must pose no danger to user health
Comfort	The EEG cap should be comfortable to wear for several hours
Mobility	To ensure mobility, the BCI system should be wireless, lightweight, and compact
User-friendliness	The system should be simple to operate and need no expert help for daily use
Cost-effectiveness	The price should be affordable for all kinds of users

### Issues in Specific EEG-Based BCI Applications

The use of P300 in a BCI wheelchair has numerous drawbacks, for example, when a patient suffers from a neural disease, specifically ALS (Bashashati et al., 2007a; Kodi et al., 2013). Usually, BCIs that use a P300 modality have a poor ITR. Additionally, several studies have stated in their findings that performance may drop after a long period owing to the reduction of the P300 amplitude due to familiarization (Iturrate et al., 2009a; Choi, 2012; Amiri et al., 2013). In a real-time system, the user needs to concentrate on the mission without interruption (Iturrate et al., 2009b), which is another weakness of such a scheme. If users focus on such visual stimuli for a significant period, they feel fatigued or suffer from sore eyes (Puanhvuan and Wongsawat, 2012; Chai et al., 2014). Therefore, such physiological control signals are neither suitable nor effective for wheelchair operation. Despite the advantages of using navigation systems that assist the control of the wheelchair with shared control, limited findings have been reported. However, it is worth noting that several cases, for instance, wheelchair operation in a corridor or a free area with unfamiliar impediments, need to be addressed. In such cases, a complete wheelchair control system with its main navigation components including mapping, localization, path planning, and obstacle avoidance is highly recommended (Widyotriatmo and Suprijanto, 2015).

With regards to BCI cursor control, only three articles have reported a success rate of above 90% (Long et al., 2012b). This information somewhat suggests that an insufficient number of experiments have yet been carried out in this area. Therefore, more experiments are needed to explore the exact limitation behind the low success rate. Although MI, P300, and hybrid approaches (MI+P300, MI+SSVEP) have been used in BCI cursor control, it was observed that the hybrid approach yields the best results (Long et al., 2012b). However, it is too early to remark on which hybrid strategy is optimal for multidimensional cursor control, and, hence, more experiments are needed to evaluate efficacy. Different factors should be taken into consideration to make a robust system, such as literacy rate, target size, timeout interval, user preferences, volume of electrodes, training period, invasiveness, and movement time.

Most of the previous studies of BCI spellers have been carried out utilizing the P300 modality owing to its credibility among researchers. Hence, there are more development opportunities in the other BCI paradigms, for example, MI, SSVEP, and hybrid. Almost all of the studies employed mean accuracy and ITR to evaluate their experiments. Nonetheless, it is noteworthy to mention that amongst a selected paradigm, the performance of one study varies from another owing to the utilization of different experimental resources. The aforesaid resources include EEG caps, electrodes, GUI, software, and data from healthy or disabled subjects, as well as the number of subjects. Hence, for conducting a comparison between different BCI spellers, the first step should be that the identical hardware and software must be used. Hitherto, to operate a BCI speller, users require expert help to set up electrodes at specific positions. Portability is another challenge to the current BCI spellers. Typing errors are another issue, as it necessitates a correction period and influences the spelling rate. Hence, further enhancements should be made to provide faster, accurate, and user-friendly spellers.

Some constituents, including data preprocessing strategy, classification model design, have a considerable impact on the performance of BCI-based person identification. Although a variety of features and classifiers have been employed to figure out the superior approach, the optimum technique has not been explored. Hence, more experiments should be carried out to identify the most appropriate technique. Moreover, more attention should be given to removing artifacts from EMG, EOG, and ECG. In an EEG-based authentication system, different paradigms like P300, SSVEP, and MI can be used, although each paradigm has its own merits and demerits. Thus, the best paradigm for EEG should be identified based on the person's authentication. Moreover, the EEG acquisition protocols should be user-friendly. Brain activity acquisition for biometric usage is a crucial matter that has not received enough attention from researchers (Campisi and La Rocca, 2014). Few partial studies have been conducted, and these studies emphasized session-wise EEG stability. Moreover, in these studies, the variation in data acquisition time lay between 1 week and 5 months (La Rocca et al., 2013; Lee et al., 2013; Palaniappan and Revett, 2014). Additionally, the human brain is very sensitive to emotion (Marcel and Millan, 2007; Lee et al., 2013). No publication has clarified the stability of EEG regarding the emotional diversity of BCI-based biometrics. Hence, the emotional diversity of the human mind should be carefully considered for a realistic BCI biometric system. Moreover, permanence in terms of elicitation strategy, applied protocol, and feature selection should also be investigated critically.

Researchers have attempted to recognize human emotion or mental state using EEG. However, there are still many challenges in this particular research domain. The EEG signal is very weak and is easily disturbed by external factors, such as subjects' movements and environment noise. Noise-free and accurate EEG is the greatest challenge in recognizing emotional states. The way in which emotions are evoked contributes significantly in emotion recognition systems. There are numerous methods of stimulation, i.e., pictures, video clips, music, memories, self-induction, environmental elicitation like light, humidity, and temperature, and games, amongst others. By using good and strong stimulation, emotion recognition is more likely to be performed with better results and higher accuracy. A number of emotion types also sometimes affect the system's performance. Another crucial matter that significantly influences the performance of the system is the signal processing platform, which includes data denoising, filtering, suitable feature set selection, and classification model design. A variety of feature extraction and classification algorithms have been carried out by the researchers, but it is still difficult to identify which model is optimal. However, PSD and SVM have been most widely used for feature extraction and classification, respectively.

Most BCI games demonstrate very low accuracy and speed as compared to conventional interfaces, suggesting that there are issues that must be addressed to facilitate the acceptance of BCI games. One of the most prevalent issues is the selection of the EEG control signal. Among SSVEP, P300, and MI, the P300 paradigm is favored by researchers for BCI-based games. With regards to tagging problems and hardware impediments, an optimal setup should be employed, for instance, an EEG headset, VR machine, and motion sensor can be integrated, and this joint system may able to trace the rotation together with the position of the targeted users (De Vos et al., 2014). A number of notable BCI-based games have been recommended in Marshall et al. (2013). Although the P300 modality has been employed to good effect in puzzle games, this system may be upgraded by motion-based technology. A turn-based strategy allows the users to pick options at their own pace, whereas a variety of simulation and adventure games are more relevant for a BCI+VR game. The performance of real-time BCI with moving users is a controversial issue. When the users are walking or moving, the P300 modality can be stimulated, but the overall performance drops (Debener et al., 2012). A camera and accelerators can be used for identifying user movement as well as removing the corresponding EEG signal from the analysis (Ahn et al., 2014). To ensure noise-free EEG, a real-time artifact elimination strategy has been recommended (Barachant et al., 2013). Additionally, some novel features, including the weighted phase lag index, can be employed when the subjects are walking (Lau et al., 2012). However, we have noticed that there is still a lack of studies outside of a controlled environment, such as Debener et al. (2012).

A limited number of studies have reported on the use of BCI in robotic arm control, suggesting that there is more room for exploration. Moreover, it is apparent that the performance of these reported systems is still at an unsatisfactory level, indicating that more experiments should be carried out. Due to a lack of necessary adaptation and training, the time taken for such activity to transpire is somewhat lengthened; nonetheless, this problem can be mitigated through proper training. Some BCI-based home appliances may able to handle only one application a time, which does not reflect effectiveness in real-life scenarios. Hence, the activities of daily living (ADL) of paralyzed patients will be easier when many home appliances at a time can be utilized through an independent system. Most of the proposed environmental control systems have been assessed on healthy users. Thus, there is a crucial question as to whether the performance of healthy users is identical to that of disabled people. Until now, the possibility of the use of environmental control systems by severely disabled people has been investigated in very few studies, and, hence, more effort should be applied to evaluating data obtained from disabled patients.

## Conclusion

A thorough review analysis has been carried out in this article on EEG-based BCI, particularly to investigate its methodological advantages and disadvantages and the essential contributions required in this field of research. Each BCI application has been explored in terms of data acquisition technique, control signals, EEG feature extraction, classification methods, and performance evaluation metrics. Finally, potential complications with EEG-based BCI systems have been addressed, and promising alternative options have been recommended. Patients with CNS injury may be able to rehabilitate their motor function through the progress of emerging BCIs. Owing to wireless recording, portability of EEG headsets, cost-effective amplifiers, significant temporal resolution, and proficient signal analysis strategies, there is keen attention on EEG-based BCI technology for such purposes. In spite of the many outstanding breakthroughs that have been achieved in BCI research, some issues still need to be resolved. First of all, a general BCI standard is currently the main issue. The BCI community should declare a general BCI standard that must be adhered to by BCI researchers. Secondly, the existing BCIs offer somewhat poor ITR for any type of effectual BCI application. Hence, future research should concentrate on increasing the ITR of BCI systems. Moreover, matching the most relevant EEG control signal with the intended BCI application is another important issue in EEG-based BCI research. Owing to the present accessibility of computational resources, researchers have begun to move away from conventional machine learning models to deep learning approaches. The use of such contemporary techniques allows for the classification of non-stationary EEG. Most of the studies on BCI have used different evaluation metrics on their own as per their convenience without any uniformity, which makes it difficult to choose the most efficient method, especially for new researchers in this field. Hence, it could be of interest to establish standardized BCI metrics so that all related works may follow the standard. These metrics would be formed based on BCI application or EEG modalities. Through this, a fair comparison could be made between related works accurately and conveniently, which would help new researchers to pursue their intended research direction. Finally, the majority of BCI applications are at the investigative phase, and they are not readily available for the ADL of the general populace. In addition, the lack of commercialization of BCI technology is also partly responsible for impeding its popularity around the world. If the abovementioned concerns can be addressed, BCI systems could be an emerging means of human-machine interaction in the foreseeable near-future.

## Author Contributions

MR and NS: conceptualization. MR and BB: methodology. MR: investigation and writing—original draft preparation. MR, AA, and BB: resources. MR, AP, RM, BB, and SK: writing—review and editing. NS: supervision and project administration. NS, AP, AA, RM, and SK: funding acquisition.

## Conflict of Interest

The authors declare that the research was conducted in the absence of any commercial or financial relationships that could be construed as a potential conflict of interest.

## Footnotes

^1^*Top 14 EEG Hardware Companies [Ranked]*. Available online at: https://imotions.com/blog/top-14-eeg-hardware-companies-ranked/ (accessed February 03, 2019).

^2^*Companies - BNCI Horizon 2020* Available online at: at: http://bnci-horizon-2020.eu/community/companies (accessed February 02, 2019).
